# A comparative study of endoderm differentiation in humans and chimpanzees

**DOI:** 10.1186/s13059-018-1490-5

**Published:** 2018-10-15

**Authors:** Lauren E. Blake, Samantha M. Thomas, John D. Blischak, Chiaowen Joyce Hsiao, Claudia Chavarria, Marsha Myrthil, Yoav Gilad, Bryan J. Pavlovic

**Affiliations:** 1 Department of Human Genetics, University of Chicago, Chicago, IL USA; 2 Department of Medicine, University of Chicago, Chicago, IL USA; 3 Cummings Life Sciences Center, 920 E. 58th Street, CLSC 317, Chicago, IL 60637 USA

**Keywords:** Comparative genomics, Functional genomics, Gene expression

## Abstract

**Background:**

There is substantial interest in the evolutionary forces that shaped the regulatory framework in early human development. Progress in this area has been slow because it is difficult to obtain relevant biological samples. Induced pluripotent stem cells (iPSCs) may provide the ability to establish in vitro models of early human and non-human primate developmental stages.

**Results:**

Using matched iPSC panels from humans and chimpanzees, we comparatively characterize gene regulatory changes through a four-day time course differentiation of iPSCs into primary streak, endoderm progenitors, and definitive endoderm. As might be expected, we find that differentiation stage is the major driver of variation in gene expression levels, followed by species. We identify thousands of differentially expressed genes between humans and chimpanzees in each differentiation stage. Yet, when we consider gene-specific dynamic regulatory trajectories throughout the time course, we find that at least 75% of genes, including nearly all known endoderm developmental markers, have similar trajectories in the two species. Interestingly, we observe a marked reduction of both intra- and inter-species variation in gene expression levels in primitive streak samples compared to the iPSCs, with a recovery of regulatory variation in endoderm progenitors.

**Conclusions:**

The reduction of variation in gene expression levels at a specific developmental stage, paired with overall high degree of conservation of temporal gene regulation, is consistent with the dynamics of a conserved developmental process.

**Electronic supplementary material:**

The online version of this article (10.1186/s13059-018-1490-5) contains supplementary material, which is available to authorized users.

## Background

Differences in gene regulation between humans and other primates likely underlie the molecular basis for many human-specific traits [1]. For example, it has been hypothesized that human-specific gene expression patterns in the brain might underlie functional, developmental, and perhaps cognitive differences between humans and other apes [2, 3]. Providing a measure of support for this notion, a recent comparative study that explored the temporal dynamics of gene regulation found potential differences in the timing of gene expression in the developing brain across primates [4]. The authors argued that such differences might be related to inter-species differences in the timing of developmental processes.

Other comparative studies of gene regulatory phenotypes in primates have resulted in important insights into the evolution of gene expression levels and the traits they are associated with [5]. Yet, we are also finding that comparative studies of gene expression patterns alone—without additional context or perturbation—provide relatively little insight into adaptive phenotypes. To a large degree, the challenge is that comparative studies in primates are extremely restricted because we only have access to a few types of cell lines and to a limited collection of frozen tissues [5]. Frozen post-mortem tissues are not optimal templates for many functional genomic assays; as a result, we lack datasets that survey multiple dimensions of gene regulatory mechanisms and phenotypes from the same individuals [5, 6]. Moreover, because it is rare to collect a large number of tissue samples from the same donor (particularly in non-human primates), we have never had the opportunity to study cross-species, population-level patterns of gene regulation in multiple tissues or cell types derived from the same genotype (same donor) in non-human apes.

Recent technological developments in the generation and differentiation of induced pluripotent stem cells (iPSCs) now provide a renewable, staged, and experimentally pliable source of terminally differentiated cells. Utilizing time course differentiation protocols, we can examine the context-dependent nature of gene regulation, as well as the temporal roles of gene expression as different cell types and developmental states are established [7]. This approach seems promising and, indeed, a handful of recent studies have been successful in utilizing iPSCs from humans and chimpanzees to characterize the uniquely human aspects of craniofacial development [8] and cortex development [9, 10].

Primate iPSC panels are a particularly attractive system for comparative studies of early development. Based on studies in model organisms, we expect differentiation into a germ layer in a mammalian system to be extremely conserved [11]. We thus set out to ask whether we can recapitulate a conserved early developmental gene regulatory trajectory in human and chimpanzee iPSC-derived cell lineages. Our rationale is that if we can demonstrate the fidelity of the iPSC model in this context, it would provide support for the notion that the iPSC system can be a useful tool for future comparative studies of dynamic biological processes.

To this end, we chose to differentiate iPSCs from human and chimpanzee into the endoderm germ layer, from which essential structures in the respiratory and digestive tracts are ultimately derived. These structures include the liver, the pancreas, the gall bladder, the lung, the thyroid, the bladder, the prostate, most of the pharynx, and the lining of the auditory canals and the larynx [11]. Using this system, we found evidence to support developmental canalization of gene regulation in both species, 24 h after differentiation from an iPSC state.

## Results

### Study design and data collection in the iPSC-based system

**Fig. 1:**
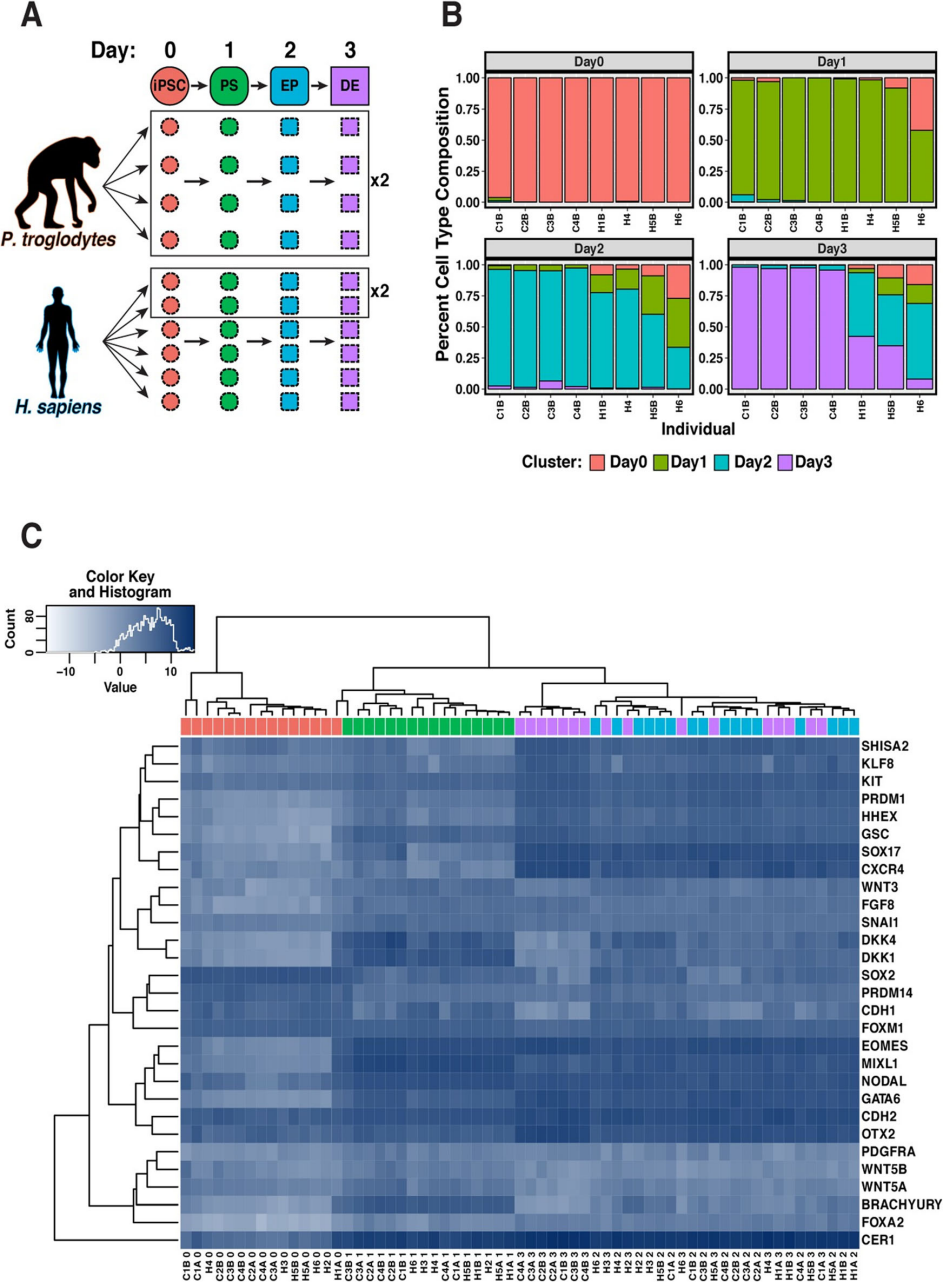
Study design and quality control analyses. **a** Study design. Samples from four chimpanzees and six humans were studied at four time points during endoderm development. We included two technical replicates from each of the chimpanzees and two technical replicates for two of the six humans. iPSC induced pluripotent stem cell, PS primitive streak, EP endoderm progenitor, DE definitive endoderm. **b** Purity analysis. Cell type composition at each day based on FACS analysis (see “Methods”), estimated by k-means clustering. **c** *Heat map* of normalized log_2_(CPM) as a measure of expression levels of TFs that are known to be highly expressed in one or more stages in the differentiation to endoderm [7]. Generally, samples from the same day, regardless of species, cluster together

To perform a comparative study of differentiated cells, we used a panel of six human and four chimpanzee iPSC lines previously derived and characterized by our lab [12, 13]. We differentiated the iPSCs into definitive endoderm, a process that was completed over three days [7], and included replicates of cell lines that were independently differentiated (see “Methods”; Fig. 1a). We performed the differentiations in two batches, with equal numbers of human and chimpanzee samples in each batch. We harvested RNA from iPSCs (day 0) before differentiation and subsequently every 24 h to capture intermediate cell populations corresponding to primitive streak (day 1), endoderm progenitors (day 2), and definitive endoderm (day 3). Overall, we collected a total of 32 human samples and 32 chimpanzee samples (Fig. 1a). We confirmed that RNA from all samples was of high quality (Additional file 1: Table S1; Additional file 2: Figure S1) and subjected the RNA to sequencing to estimate gene expression levels. Detailed descriptions of all individual donors, iPSC lines, sample processing and quality, and sequencing yield can be found in the “Methods” section and Additional file 1: Table S2A and B.

**Fig. 2:**
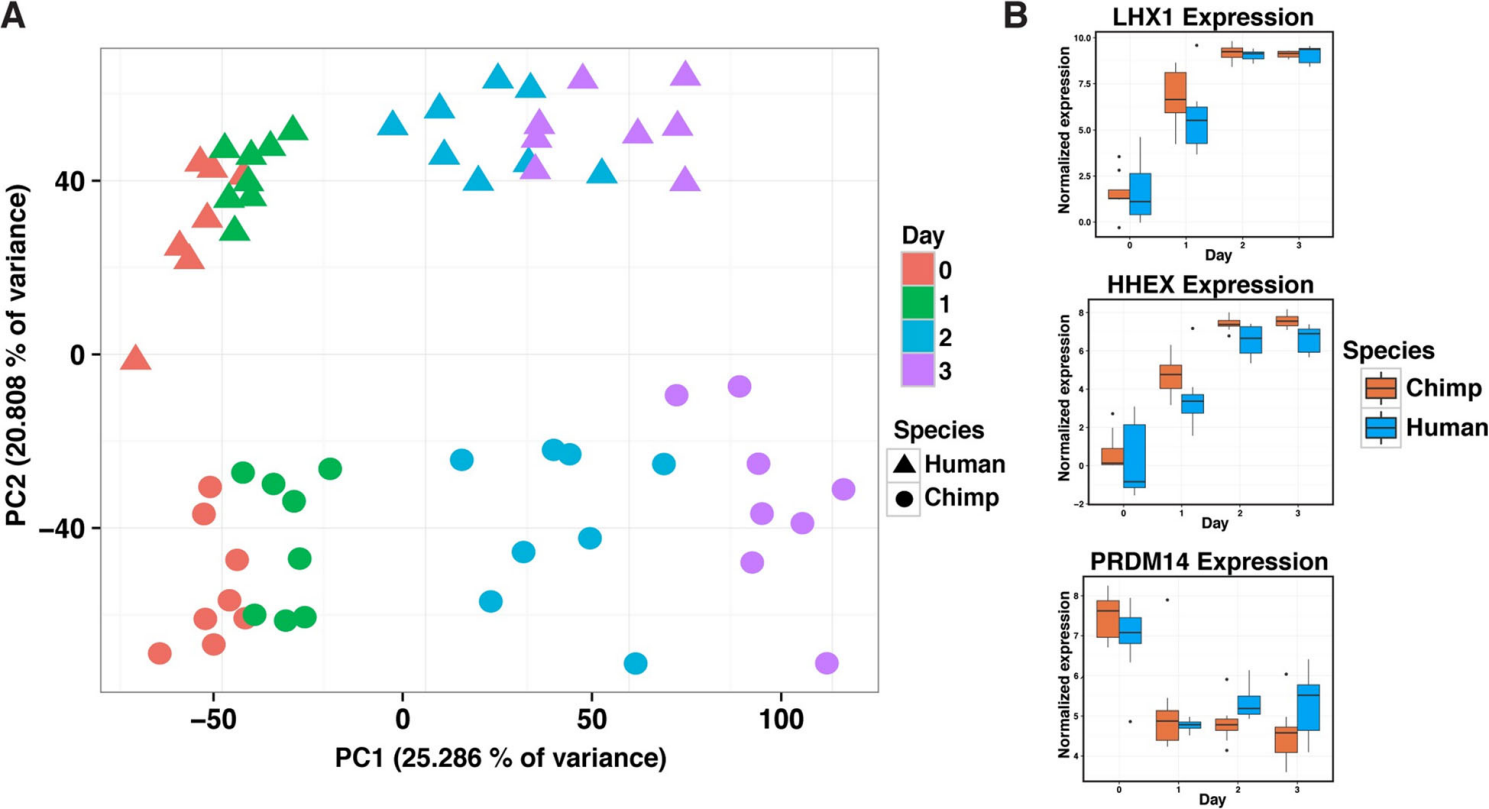
General patterns in the data. **a** Normalized log_2_(CPM) expression measurements for all genes projected onto the axes of the first two PCs. *Color* indicates day. *Shape* represents species. PC1 is highly correlated with differentiation day (*r* = 0.92). PC2 is highly correlated with species (*r* = 0.93). **b** *Box plots* of normalized expression values for genes with known roles in endoderm development

To estimate gene expression levels, we mapped reads to the corresponding genome (hg19 for humans and panTro3 for chimpanzees) and discarded reads that did not map uniquely [14]. We then mapped the reads to a list of previously described metaexons across 30,030 Ensembl genes with one-to-one orthology between human and chimpanzee [6, 15]. We eliminated genes that were lowly expressed in either species, removed data from one clear outlier sample (H1B at Day 0; Additional file 2: Figure S2A), and normalized the read counts (see “Methods”; Additional file 2: Figure S2B and C) to obtain TMM- and cyclic loess-normalized log_2_ counts per million (CPM) values for 10,304 orthologous genes (Fig. 2a; Additional file 3). These normalized gene expression values were used in all downstream analyses.

Beyond the gene expression data we collected, in the second batch, we assessed the purity of the cell cultures at each day of the time course using flow cytometry with a panel of six canonical markers. These markers correspond to the cell types we expected in the different stages of differentiation (Fig. 1b; Additional file 2: Figure S3A and B). We also assessed the purity of the samples in the first batch of differentiation; however, due to a technical problem with the antibodies, those values are not informative. Overall, the FACS-based estimated purity levels for the sample in the second batch are high and consistent across species in days 0 and 1 (> 0.79 and > 0.66, respectively; Fig. 1b; Additional file 1: Table S3; Additional file 2: Figures S3 and S4). On days 2 and 3, however, purity levels were considerably lower in the human than in the chimpanzee samples (Fig. 1b; Additional file 1: Table S3; Additional file 2: Figures S3 and S4). On the one hand, this inter-species difference in purity in the later stages of the time course limits the insight we can draw from this comparative study, as we discuss throughout the paper. On the other hand, because our main focus is to confirm that this early developmental process is conserved in the two species, the observation of strong conservation, which we discuss below, is robust with respect to this inter-species technical difference in purity.

### iPSCs-based system effectively models primate endoderm differentiation

Given the potential impact of study design properties on gene expression data and subsequent conclusions [16], as a first step of our analysis, we confirmed that none of our recorded variables related to sample processing (apart from sample purity, as stated above) were confounded with our main variables of interest, namely day and species (see “Methods”; Additional file 1: Table S4A–D; Additional file 2: Figures S2C and S5). Once we were confident that our study design provided an effective dataset for addressing our biological questions of interest, we performed a global survey of the gene expression data using principal component analysis (PCA). This analysis indicated that the primary sources of gene expression variation are differentiation day (Fig. 2a; Additional file 1: Table S4A–E; regression of PC1 by differentiation day, *P* < 10^−15^), followed by species (regression of PC2 by species, *P* < 10^−15^). This observation was also supported by clustering analysis based on the correlation matrix of pairwise comparisons of the gene expression levels (Additional file 2: Figure S6).

After characterizing global gene expression patterns, we focused on the expression of specific transcription factors (TFs) with known roles in developmental pathways (Fig. 1c) and other previously known lineage-specific markers [7, 17, 18]. Consistent with the results of our FACS analysis, we observed that the temporal trajectory of expression levels of known lineage-specific markers and TFs further supported the assumed differentiation stages in each day (e.g. primitive steak-specific markers had increased expression on day 1, Fig. 2b). The lineage-specific markers and TFs were expressed at comparable levels in humans and chimpanzees at the relevant time points, consistent with previous literature [7, 17], and further supporting the validity of our in vitro system (Additional file 2: Figure S7A).

### Comparative assessment of gene expression changes during differentiation

**Fig. 3:**
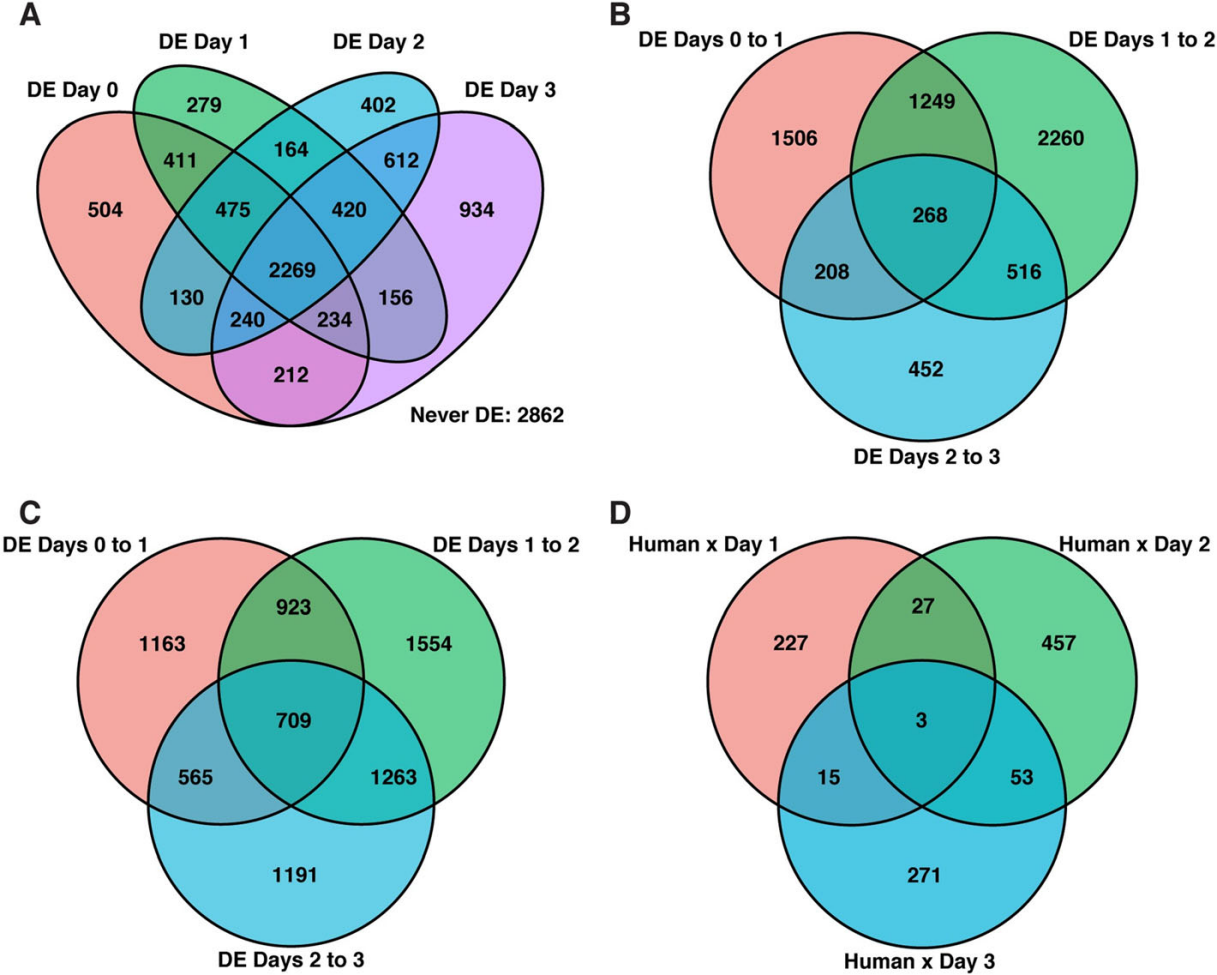
Number of DE genes in pairwise analyses. *Venn diagrams* of **a** DE genes at each day, **b** DE genes between consecutive time points in humans, **c** DE genes between consecutive time points in chimpanzees, **d** genes with a significant species–time point interaction effect at each day (DE was classified at FDR of 5% in all cases)

To identify gene expression differences between humans and chimpanzees throughout the time course, we used the framework of linear models (see “Methods”). We first assessed how many genes were differentially expressed (DE) between species at each time point independently. Using this approach, we classified thousands of genes as DE between the species (at FDR of 5% 4408–5077 genes are classified as inter-species DE at different times points; Fig. 3a; Additional file 1: Table S5A–D). Even at a fixed FDR cutoff, nearly half of the genes that were classified as DE between the species at any single time point were found to be DE in all time points (2269 genes). Nearly one-third of genes whose expression was measured in our experiment were not classified as DE between the species at any time point (2862 genes, 28%).

**Fig. 4:**
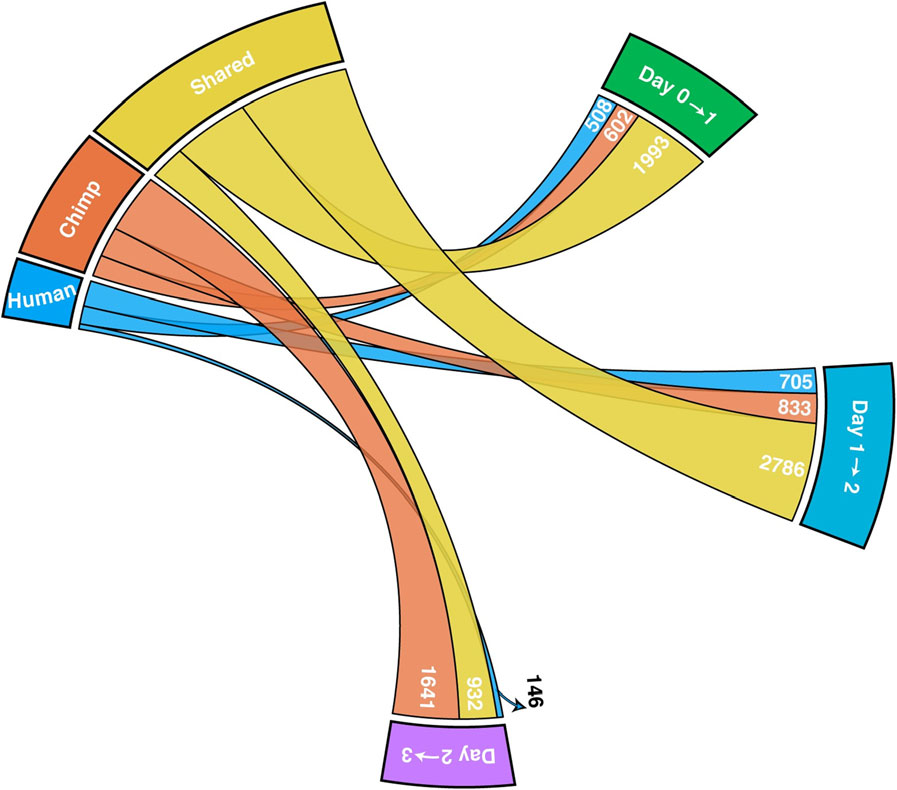
High sharing of DE genes across species. A *circos diagram* with the number of shared, human-specific, and chimpanzee-specific DE genes across time points. There is a high degree of sharing of DE genes (yellow ribbon), particularly from day 0 to 1 and day 1 to 2

We proceeded to consider temporal expression patterns within species. When analyzing expression changes across consecutive time points, we had more power to detect temporal gene expression differences in chimpanzee compared to humans (Figs. 3b, c and 4; Additional file 1: Table S6A–F), especially with respect to the transition between endoderm progenitors (day 2) and definitive endoderm samples (day 3). This property is likely related to the inter-species difference in purity of samples from these days, as discussed earlier. When we accounted for incomplete power (see “Methods”), we found a remarkably consistent pattern whereby 77% of DE genes between iPSCs and primitive streak in humans are also DE between these states in chimpanzees; similarly, 77% of DE genes between primitive streak and endoderm progenitors in humans are also DE between these states in chimpanzees; and 80% of DE genes between endoderm progenitors and definitive endoderm in humans are also DE between these states in chimpanzees (Additional file 1: Table S7). As might be expected from these observations, we found that the relationship between day and gene expression was largely independent of species (Fig. 3d; Additional file 1: Table S8A–C).

### Joint Bayesian analysis reveals conservation of temporal gene expression profiles

In an attempt to further overcome issues of incomplete power affecting these original naïve pairwise DE comparisons, and to account for dependency in data from different time points, we utilized a Bayesian clustering approach implemented by Cormotif [19]. This joint modeling technique leverages expression information shared across time points to identify the most common temporal expression patterns (referred to as “correlation motifs”).

**Fig. 5:**
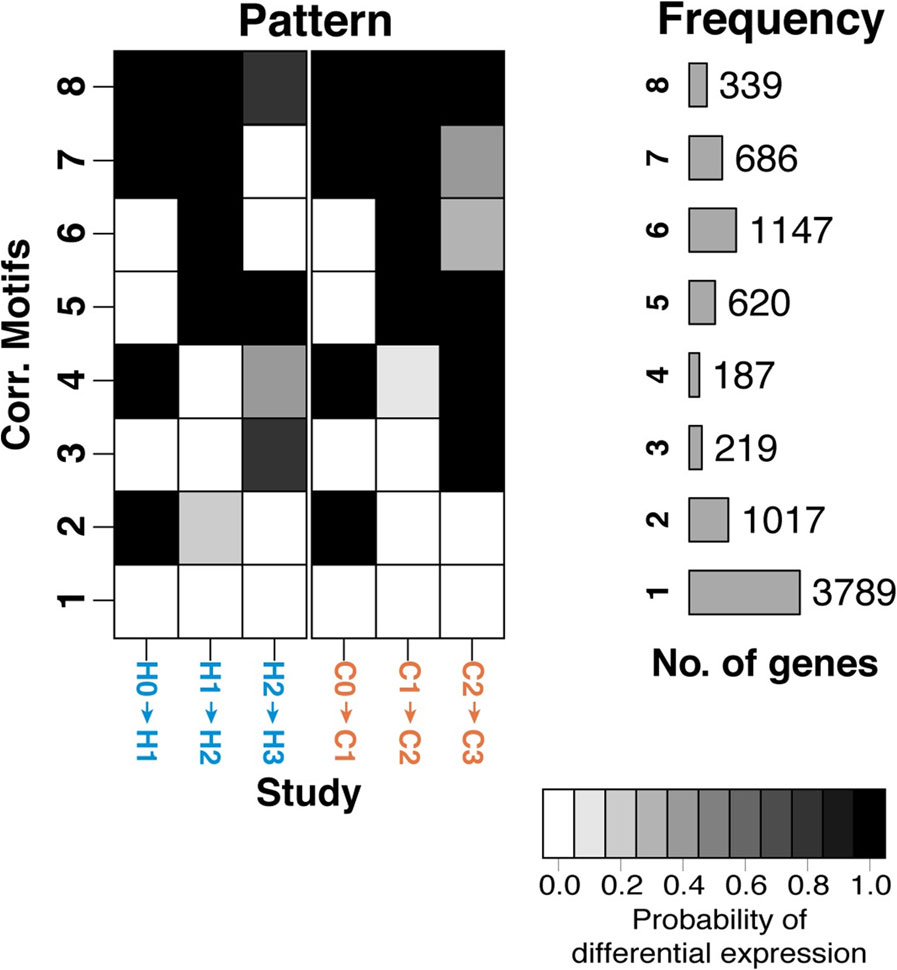
Gene expression motifs. Correlation motifs based on the probability of differential expression across days for each species with the number of genes assigned to each correlation motif. The shading of each box represents the posterior probability that a gene is DE between two time points in a given species. Each row (“correlation motif”) represents the most prevalent expression patterns. Out of 10,304 genes, 8004 were assigned to one correlation motif in this model

We identified diverse expression patterns that emerge as differentiation progresses in both species (Fig. 5; Additional file 1: Table S9A) as well as a set of 3789 genes whose expression is not significantly altered throughout the time course (8004 genes could be reliably classified into a motif, Additional file 1: Table S9A; Additional file 2: Figure S8A). This analysis revealed further evidence for conserved gene expression patterns, as 75% of genes assigned were assigned to motifs with the same or similar temporal regulatory trajectories in both species (Fig. 5). Further, when we excluded data from the definitive endoderm samples, where we suspect that a particularly large inter-species difference in sample purity has increased gene expression variance between the species, we assigned 85% of genes to motifs with the same temporal trajectories across species. These observations are robust with respect to the number of correlation motifs, the method used to combine data from technical replicates, which days were included in the pairwise comparisons, and the inclusion of all 10,304 genes in the analysis (Additional file 2: Figures S8B–D and S9; Additional file 4). Our observations are also robust with respect to the overall approach used to estimate and compare gene expression trajectories (Additional file 2: Figure S7B; Additional file 4).

We found two correlation motifs with a potential marked difference between the species at a given stage (motif 4 with 187 genes and motif 7 with 686 genes). In both of these motifs, data from the earliest time points were conserved but gene regulation in the final stage (days 2–3) differed between the species. The genes in these motifs were enriched for Gene Ontology (GO) annotations related to animal organ development (e.g. *NRTN*, *PITX2*, *RDH10*), anatomical structure morphogenesis (*ARHGDIA*, *EHD2*, *SERPINE1*), regulation of developmental process (*FLRT3*, *LOXL2*, *SEMA7A*), and regulation of cell differentiation (*DIXDC1*, *ENC1*, *IRF1*; Additional file 1: Table S9B) [20]. These four GO annotations were not enriched in other similarly sized motifs or group of motifs (Additional file 1: Table S9C–E) [20, 21]. Unfortunately, due to interspecies differences in purity at the later days, we cannot definitively determine whether these enrichments are driven by biological or technical differences.

### Reduced variation in gene expression levels at primitive streak

We next turned our attention to differences in the magnitude of variation in gene expression levels across time points, within and between species. Previous studies reported that variation in gene expression levels between individuals was lower in iPSCs than in differentiated cells ([22, 23] and Additional file 2: Figure S10A). We were thus interested in gene expression variation during iPSC differentiation in our comparative system.

**Fig. 6:**
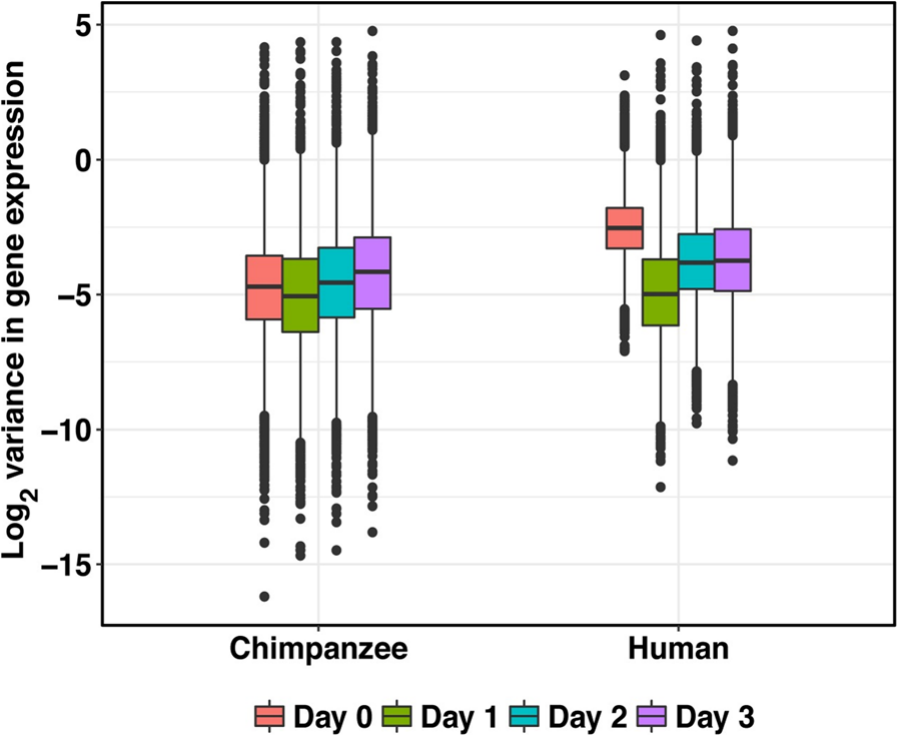
Global reduction of variation in gene expression from the iPSCs to primitive streak state. *Box plot* of the log_2_ variance of expression levels for each gene. Variation in gene expression levels are significantly reduced from iPSCs to primitive streak (*P* < 10^−15^ in both species) but not in subsequent time points (*P* > 0.5 in both species)

We first compared within-species expression variation for all 10,304 orthologous genes across time points. We found a reduction in inter-individual variation of gene expression levels as the human samples differentiated from iPSCs to primitive streak (*P* < 10^−15^, Fig. 6). We also detected this pattern when we considered the chimpanzee samples (*P* < 10^−15^, Fig. 6), but the effect size in chimpanzee is much smaller. We did not identify a similar reduction in variation in gene expression levels in any other transition during the time course in either species (*P* > 0.5 for testing the null of no change in variance of gene expression from day 1 to day 2 and from day 2 to day 3 in each species). As mentioned above, the purity of the samples in days 2 and 3 is lower than that of samples in days 0 and 1, though we only successfully measured the purity values for the samples that were processed in the second batch. We accounted for the measured purity values by regressing them out of the gene expression data. As might be expected, accounting for the purity values resulted in different patterns observed for the data from days 2–3. Yet, the reduction in variation from day 0 to day 1 persisted in both species even after we accounted to the purity values (Additional file 2: Figure S10B).

We turned our attention to regulatory divergence between species. The overall human–chimpanzee divergence in gene expression levels was also slightly reduced as samples differentiated from iPSCs to primitive streak (Mann–Whitney *U* test, *P* = 0.04; Additional file 2: Figure S10C), but not in any other transition during the time course. Furthermore, while we classified 504 genes as DE between humans and chimpanzees exclusively in iPSCs (of a total of 4475 DE genes in iPSCs, FDR = 5%; Fig. 3a; Additional file 1: Table S10), we found only 279 genes that were DE exclusively in primitive streak samples (from a total of 4408 DE genes for the primitive streak; Fig. 3a; Additional file 1: Table S10). The number of genes that are DE between the species exclusively in endoderm progenitors and definitive endoderm samples is higher (at FDR of 5%, 402 and 934, respectively; Fig. 3a; Additional file 1: Table S10). The observation of a smaller number of genes that are DE exclusively in primitive streak samples compared with iPSCs is robust with respect to normalization method, purity of the samples, FDR cutoff, and differentiation batch (Additional file 1: Table S10; Additional file 2: Figures S2C, 10C, and S11). While the difference in divergence and the number of DE genes between these differentiated states is modest and could potentially be explained by a number of non-biological factors (including the differences in purity in the later day), this observation was intriguing to us.

We thus focused on the transition between iPSCs to primitive streak in both species. The recorded technical factors (including purity for these states) are highly similar across biological conditions in days 0 and 1, and therefore are not likely to explain this observation (Additional file 1: Table S11). We thus proceeded to analyze the trajectory of variation in expression level on an individual gene basis. In this analysis, we were particularly interested to address whether the individual genes that undergo a change in variation of expression levels are shared across species. An observation of excess sharing could not be explained by inter-species differences in technical factors and hence would provide substantial support to the notion of conserved reduction of regulatory variation as the samples begin their differentiation process.

**Fig. 7:**
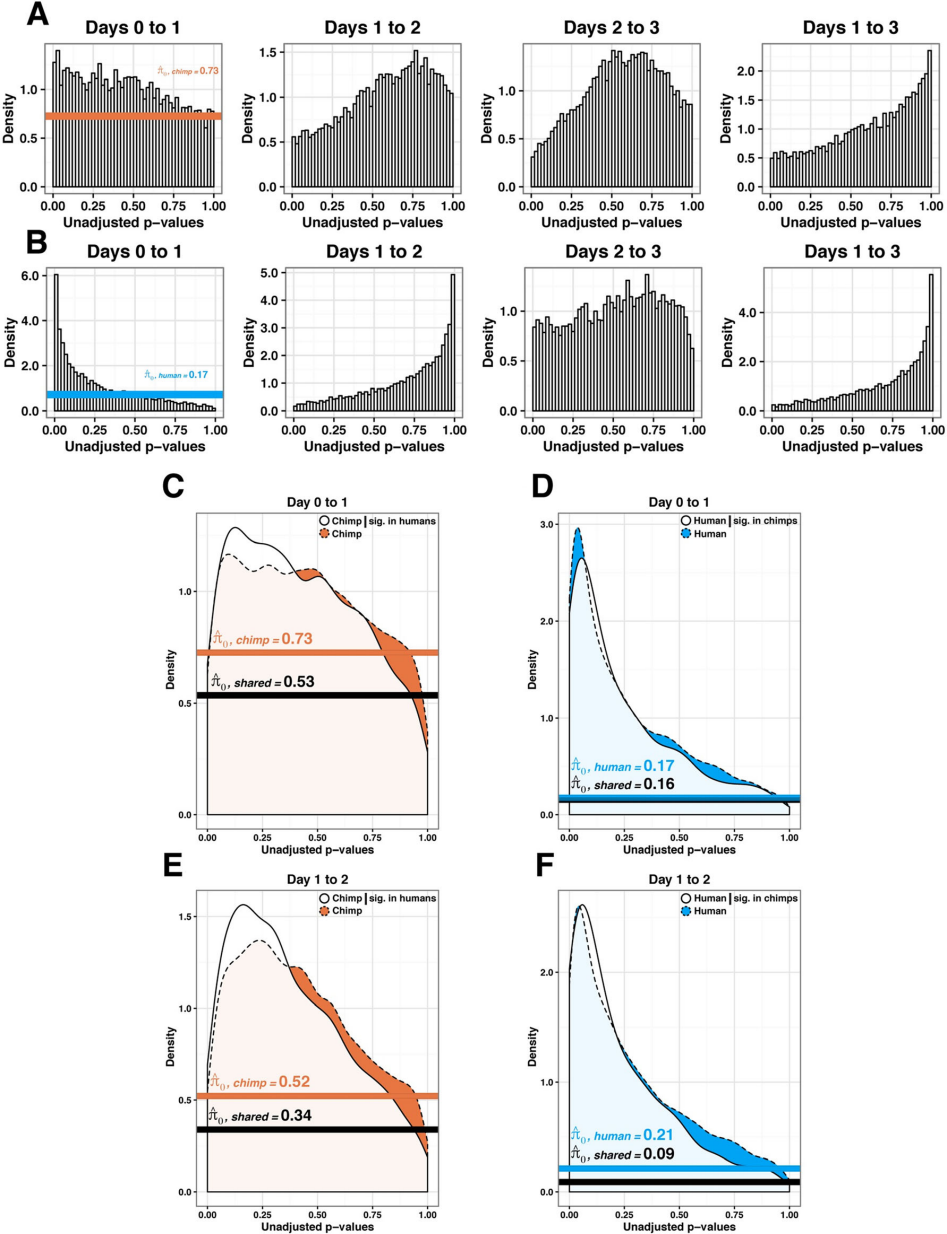
Conserved patterns of reduced variation in gene expression at primitive streak. We plotted the *P* value distributions of F tests of the null hypothesis that there is no reduction in variation in gene expression levels as samples progress along the time course in human (**a**) and chimpanzee (**b**) samples. $\widehat{\uppi}$_0_ is the estimated proportion of null tests in each distribution. In the next four panels, we plotted the *P* value distribution for the same test, but included only genes whose variation was classified as reduced between states in the other species; thus in (**c**) we plotted *P* value distributions of F tests in chimpanzees only for genes who variation was classified as reduced (*P* < 0.05) in humans and in (**d**) we did the reverse. In (**e**) (chimpanzee conditional on human) and (**f**) (human conditional on chimpanzee), we plotted the *P* value distributions of F tests of the null hypothesis that there is increase in variation in gene expression levels as the samples progress from day 1 to 2

We used F tests to identify genes whose within-species variation in expression levels differs across time points (see “Methods”). Distributions of *P* values from all tests can be found in Fig. 7a and b and Additional file 1: Table S12A and B, which indicate that for a large number of genes, within-species variation in expression levels were reduced exclusively in primitive streak samples. Indeed, while we did not have much power to detect differences in variation of individual gene expression levels between states (due to the small number of individuals in each species), we observed a clear excess of small *P* values when we tested the null hypothesis that there was no reduction in gene expression levels from day 0 to day 1. Using Storey’s approach [24] to account for incomplete power, we estimated that within-species variation in expression levels was reduced as the samples differentiate from iPSCs to the primitive streak in 83% and 27% of human and chimpanzee genes, respectively (Fig. 7a and b). This result was robust with respect to the method used to calculate the proportion of true positives [25] (Additional file 2: Figure S12). We did not observe reduced variation of gene expression in any other differentiation state in our data (Fig. 7; Additional file 2: Figure S13A).

We next asked about the overlap of genes with reduced variation in primitive streak samples across the two species. Specifically, we asked whether human genes with lower within-species variation in expression levels in primitive streak are more likely to show the same pattern in chimpanzee genes. For this analysis, we again used the Storey approach [24] to estimate the proportion of true-positive tests in one species, conditional on the observation of reduced variation in the other species (see “Methods”). We estimated that 47% of genes whose variation in expression level is reduced in human primitive streak samples showed a similar pattern in chimpanzees (under a permuted null we expect 27%, *P* < 10^−4^, Fig. 7c; Additional file 1: Table S13). When we condition on observing a reduction of variation in chimpanzees, the overlap with humans was 84% (under a permuted null we expect 83%, *P* = 0.38; Fig. 7d). This high value was not unexpected because of the initial large proportion of human genes with a clear signature of reduced variation in primitive streak. Because any technical differences that are confounded with species would contribute to increased inter-species differences, these observations support, yet probably underestimate, the degree of high conservation of regulatory patterns in humans and chimpanzees.

Using a similar approach to account for incomplete power, we also found a marked overlap of genes whose expression underwent a significant increase in variation throughout the transition from primitive streak to endoderm progenitors (Fig. 7e, f; Additional file 2: Figure S13B). All our observations were robust to a wide range of statistical cutoffs used to classify genes whose within-species variation changes across the differentiation states (Additional file 2: Figures S14–S16). In particular, because these are reports of conserved patterns, they are likely to underestimate the degree of conservation given technical differences between the species, including the difference in purity of the samples on days 2 and 3.

Finally, we sought to provide insight into the potential functional consequences of our observations. Interestingly, genes that show reduction of variation in gene expression levels in both species are enriched for GO annotations [20] related to development, including neuroepithelial cell differentiation (e.g. *MYCL*, *NODAL*, *CDH2*), cell migration during gastrulation (*FGF8*, *MIXL1*), and trophectodermal cell differentiation (*CNOT3*, *EOMES*, *SP3*; Additional file 1: Table S14). Moreover, we found that null mutation in mouse orthologs [26] of the primate genes with shared reduction of expression variation in our study were more highly associated with embryonic lethality than null mutation in mouse orthologs of primate genes that did not show that pattern (41% vs 28%, *P* < 10^−4^ [27, 28]).

## Discussion

Our results indicate a strongly conserved temporal expression profile across species during early differentiation. We observed a large number of genes with similar expression profiles across species. Indeed, we found that DE genes between differentiation states are shared between the two species far beyond what is expected by chance alone. When we jointly analyzed data from the entire time course, nearly all the gene expression trajectory motifs we identified, including 75% of all genes assigned to a motif, are shared across the two species (Fig. 5). Still, our observations likely underestimated the proportion of shared regulatory patterns due to incomplete power.

Our finding that regulatory trajectories throughout endoderm differentiation are generally highly conserved in these two species was expected. Yet, our observation that a large number of genes are associated with conserved reduced regulatory variation in a specific transition state is a somewhat surprising property. Indeed, in our opinion, the most significant finding of this study is the observation that regulatory variation is reduced in both humans and chimpanzees as the cell cultures differentiate from iPSCs to primitive streak. We found a marked overlap between the species in the specific genes that experience this reduction of regulatory variance, indicating a high degree of conservation in this process.

Before we discuss the potential implications of our observations, we will first discuss a few considerations regarding the iPSC-based differentiation models. We argue that the use of iPSC models allows for greater control and transparency of comparative studies in primates, including a better appreciation of caveats that have always affected such studies but were typically cryptic.

For more than a decade, comparative genomics in primates has relied on the use of frozen tissues. An implicit assumption underlying the use of these tissues has been that they faithfully reflect interspecies gene regulatory similarities and differences. Yet, we know that gene regulation in these tissues was likely impacted by non-genetic factors, such as the individual’s diet, age, and cause of death. In addition, it is nearly impossible to stage frozen tissues with respect to cellular composition. In fact, in practically all studies of comparative data from frozen tissues, cellular composition was not even measured. Indeed, comparative studies of gene expression in primate frozen tissue samples, including those by our own group, simply assumed that most observed patterns are driven by genetic control.

In contrast to frozen tissues, the primate iPSC-based differentiation model allows us to minimize the impact of non-genetic (e.g. environmental) factors. We can also control to a large extent the cellular composition of the samples and, more importantly, we can measure and account for differences in cellular composition. Comparative experiments with iPSC-based differentiation are certainly not flawless, but they allow us to more explicitly characterize, and often account for, confounding factors than was possible with frozen tissue samples. In the case of the current study, we used human and chimpanzee iPSC lines that were generated, and differentiated, using the same protocols. We made considerable efforts to balance the majority of sample processing properties related to our study design with respect to species and time point. For example, the two differentiation batches we used included multiple human and chimpanzee lines (which we also balanced with respect to gender). Admittedly, in vitro differentiation protocols are not identical to natural developmental signaling and natural cell-driven developmental processes may be overridden by our administered media conditions. Moreover, although we used an identical differentiation protocol across the entire experiment, we observed a wide distribution of cell purity across samples, with a marked difference between the species in the purity of cultures from days 2 and 3.

The fact that we are able to measure and discuss purity and cellular composition as a potential flaw in our study is an advantageous property of the iPSC-based differentiation system. At present, we cannot exclude the possibility that the observed differences in cellular heterogeneity across time points may have driven the observation of reduced regulatory variation exclusively in primitive streak samples. In our opinion, however, this is unlikely, though our arguments are mostly circumstantial and we will not be able to provide a definitive answer without additional single-cell experiments (which will be the scope of a future study). Our intuition is based on the following properties: first, the iPSC cultures are the most homogenous in our experiment and we nevertheless observe a reduction of variation in gene expression levels in day 1 samples. Second, the conclusion of high sharing and similarities between species should be robust (conservative, in fact) with respect to technical differences between species, including in purity. Third, when we account for the purity values measured for the second batch of samples, the relative expression patterns remain similar.

That said, to provide a definitive answer, we will revisit these questions with an experimental design that involves single-cell data from the same comparative differentiation trajectory. Single-cell RNA-sequencing (scRNA-seq) data will also be able to shed light on our observation that gene regulation in definitive endoderm (day 3), unlike earlier days, does not indicate strong conservation between the species. In definitive endoderm samples, we observed the largest number of DE genes across species, the smallest number of DE genes between two consecutive time points in humans, and the lowest overlap in DE genes between time points. Unlike in the human samples, the chimpanzees had a relatively consistent number of DE genes between any two consecutive time points. Some of these observations might be explained by the cell purity difference between species.

It should be noted that the effect sizes for the reduction in variation from day 0 to day 1 are small in the chimpanzee samples and that the *P* value distributions do appear quite different across species. These differences, however, do not invalidate our finding that there is a significant overlap of genes that undergo a reduction of variation in gene expression levels in both species. If the reduction of variation in gene expression levels were spurious within each species, then we would not expect a statistically significant overlap of such genes. The observation of reduced regulatory variation is rather unusual in general, partly due to the unusual design of our study. Indeed, only few comparative studies have been designed to allow one to measure changes in variation over time. One such example, from a completely different context, can be found in a previous study in which monocytes from humans, chimpanzees, and rhesus macaques, which were stimulated with lipopolysaccharide (LPS) to mimic infection [29]. When comparing gene expression in LPS-stimulated monocytes to that of non-stimulated cells, the authors found a reduction of inter-species variation in gene expression levels in a number of key TFs involved in the regulation of *TLR4*-dependent pathways.

In our study, we found enrichment for genes in developmental pathways among genes with conserved reduction of expression variation. This observation is consistent with the notion [30] that developmental pathways need to be tightly regulated in general. This notion is also supported by deep conservation and lethality upon disruption seen in many of these genes. Null mutations in mouse orthologs of > 40% of the genes with conserved reduced regulatory variation at primitive streak are associated with embryonic lethality. For example, *Xenopus laevis* embryos with null mutations in the ortholog of human *MIXL1* exhibit abnormalities in primitive streak and node formation [31]. Similarly, homozygous null *MIXL1* mice have abnormal mesoendoderm development and do not survive to birth [32]. *EOMES* homozygous null mice lack trophoectoderm outgrowth [33] and do not properly form the definitive endoderm [34]. This mutation is lethal early in gestation. Overall, regulation of these genes is likely to be finely tuned at early development.

Indeed, reduced regulatory variation early in the endoderm differentiation process may be driven by the property of canalization during development. The theory of canalization posits that developmental processes end in a finite number of states despite minor environmental perturbations [30, 35, 36]. Canalization is fundamentally linked to evolutionary states [35] and thus phenotypic robustness; therefore, even when reduced variation in gene expression levels is observed in cell culture, the explanation of canalization is intuitively appealing considering the discrete nature of cell types in an adult animal. Our results suggest that stages subsequent to primitive streak may follow a more relaxed transcriptional regulation with higher influence of individual genotypes.

## Conclusions

Our observations may be consistent with activation of deeply conserved regulatory programs at the initial stages of gastrulation followed by processes less affected by evolutionary constraint and therefore potentially more amenable to adaptation. In other words, our results support the expectation that gastrulation is a highly canalized and conserved process in humans and chimpanzees. More generally, we believe that despite limitations to studying comparative development using iPSC models, which we have discussed, this system provides the opportunity to study previously unappreciated aspects of primate biology.

## Methods

### Human and chimpanzee iPSC panels

In this study, we include four chimpanzee iPSC lines (two males, two females) from a previously described panel [12] and six human lines (three males, three females) [13] matched for cell type of origin, reprogramming method, culture conditions, and closely matched to passage number (median passage was within one passage across species and differentiation batches). We evaluated iPSC lines for pluripotency measures, differentiation potential, lack of integrations, and normal karyotypes as described previously [12, 13] (Additional file 2: Figures S17 and S18). We identified one human individual (H5) that tested positive for episomal vector sequence (Additional file 2: Figure S18). This individual was not an obvious outlier in any of our data (Figs. 1b and c and 2), thus we choose to include it in our study. Original chimpanzee fibroblast samples for generation of iPSC lines were obtained from the Yerkes Primate Center under protocol 006–12. Human fibroblasts samples for generation of iPSC lines were collected under University of Chicago IRB protocol 11–0524. Feeder-free iPSC cultures were initially maintained on Growth Factor Reduced Matrigel using Essential 8 Medium (E8) as previously described. After ten passages in E8, all cell lines were transitioned to iDEAL feeder-free medium that was prepared in-house as specified previously [37]. Cell culture was conducted at 37 °C, 5% CO_2_, and atmospheric O_2_.

### Endoderm differentiation

To produce definitive endoderm and intermediate cell types, we followed a recently published three-day protocol that systematically identified and targeted pathways involved in cell fate decisions, at critical junctures in endoderm development [7] with minimal modification. At 12 h before initiating differentiation, iPSC lines at 70–90% confluence were seeded at a density of 50,000 cells/cm^2^. Basal medial for differentiations consisted of 50/50 IMDM/F12 basal media supplemented with 0.5 mg/mL human albumin, 0.7 μg/mL Insulin, 15 μg/mL holo-Transferrin, 1% *v*/v chemically defined lipid concentrate, and 450 uM 1-thioglycerol (MTG). For differentiation, basal media was supplemented with the following: day 0 to day 1 (Primitive streak induction) media included 100 ng/mL Activin A, 50 nM PI-103 (PI3K inhibitor), 2 nM CHIR99021 (Wnt agonist), days 1→ 2 (total of two media changes) media included 100 ng/mL Activin A and 250 nM LDN-193189 (BMP inhibitor). Two independent differentiation batches were performed, resulting in replicates for a subset of individuals (Additional file 1: Table S2). Each chimpanzee was replicated, while only two human individuals were replicated across the two batches. Replicates were sex-balanced both within and across species. Cell culture was conducted at 37 °C, 5% CO_2_, and atmospheric O_2_.

### Purity assessment using flow cytometry

Cells were dissociated using an EDTA-based cell release solution, centrifuged at 200 × g for 5 min at 4 °C and washed with PBS. Subsequently, 0.5–1 million cells were fixed and permeabilized using the Foxp3 / Transcription Factor Staining Buffer Set from eBioscience. Cells were fixed at 4 °C for 30 min before washing once using FACS buffer (autoMACS® Running Buffer, Miltenyi Biotech). A total of 150,000 cells were transferred to BRAND lipoGrade 96-well immunostaining plates and centrifuged at 200 × g for 5 min at 4 °C. Cells were rinsed in FACS buffer then resuspended in the staining solution. A single master mix containing 1X Permeabilization buffer (eBioscience), BD Horizon Brilliant Stain Buffer, and antibodies was prepared and 30 μL of this mix was added to each well containing cells.

In order to estimate purity for each day of the time course, we utilized a mixture of six different directly labeled antibodies: *OCT3/4* (BV421 labeled clone 3A2A20, Biolegend), *SOX2* (PerCP-Cy5.5 labeled clone O30–678, BDbio), *SOX17* (Alexa 488 labeled clone P7–969, BDbio), *EOMES* (PE-Cy7 labeled clone WD1928, eBioscience), *CKIT* (APC labeled clone 104D2, Biolegend), and *CXCR4* (BV605 labeled clone 12G5, Biolegend). All antibodies were used at the manufacturer-recommended dilution except *CKIT* and *CXCR4*, which were used at one-tenth of the manufacturer’s specified concentration (15 ng of each antibody in final volume of 30 μL per staining). We found that the manufacturer-recommended dilution produced acceptable results for live cells; however, upon fixing, we observed non-specific binding by all populations. Thus, we determined the optimal antibody titer to maximize the separation between iPSCs (biological negative) and day 3 definitive endoderm. We found this optimal concentration to be in concordance with that quantity specified by a previous publication using the same antibody clone from a different manufacturer [38]. Cells were stained for 1 h at 4 °C and subsequently washed 3× using a solution of BD Horizon Brilliant Stain Buffer containing 1X Permeabilization buffer; on the final wash, cells were resuspended in 100 μL FACS buffer for acquisition on a BD LSR II flow cytometer.

After data acquisition compensation, we used the program FlowJo (http://docs.flowjo.com/d2/credits-2/) to determine scaling. To do so, we used data from single stained compensation beads (Life Technologies) that were stained and collected in parallel. Live, intact, single cells were gated based on FSC and SSC channels as previously described [7]. Day 0 iPSC purity was estimated by dual positive *OCT3/4* and *SOX2* [39] as well as negative staining for *EOMES*. Day 1 primitive streak purity was estimated primarily based on *EOMES* Positive staining [17, 40] but also negative staining for *SOX17*. Day 2 endoderm progenitor purity was quantified by positive staining for *SOX17* expression [41] (*CKIT* could also be used, as its level peaks at day 2) and negative staining for *CXCR4*. Finally, day 3 definitive endoderm purity was estimated by double staining for *CKIT* and *CXCR4* [38]. For all time points, cells were stained with the full complement of markers; initial gates were defined using fluorescence intensity levels of an iPSC line as a biological negative control for days 1, 2, and 3. For day 0 (iPSCs), a definitive endoderm time point was used to quantify the biological negative for *OCT3/4* and *SOX2* fluorescence intensity. All iPSC lines regardless of species were at comparable fluorescence intensity levels, so we choose a representative chimp and human line to use as our standard for defining and refining all gates.

Fully resolving all time points simultaneously required us to define high and low staining gates, which were determined using the time points for that marker’s maximum and minimum fluorescence intensities. All gates were refined using the same two representative chimpanzee and human lines as used for determining biological negatives, resulting in one universal gating scheme that was applied to both species and all time points. A complete gating scheme is outlined in Additional file 2: Figure S3A, with the final purity results for the second batch of differentiation in Additional file 1: Table S3. The samples in the first differentiation batch demonstrated hallmarks of improper fixing (highly non-specific staining of antibodies, most notably for surface markers *CXCR4* and *CKIT*); thus, we were unable to determine reliable purity estimates for the first differentiation batch.

Purity was also determined using k-means clustering of minimally preprocessed flow cytometry data. After applying the same live, intact, single-cell gating scheme used above, we exported compensated fluorescence channel values for processing in R. First, we visually inspected population separation by performing PCA on compensated fluorescence channel values. We randomly sampled 1000 cells from each individual at each day, resulting in a total of 4000 randomly sampled cells per individual and 31,000 cells overall (no data were collected for individual H4 at day 3). To assign cells to a specific developmental day, we performed K-means clustering using K = 4 on a correlation matrix representing pairwise correlations between all 31,000 single cells. Four relatively well separated populations were visible after projection onto the first three principal components (PCs) (Additional file 2: Figure S4, top panel) and cluster assignment on the reduced data is shown in Additional file 2: Figure S4, middle and bottom panels. We estimated cellular composition at each day by using the PCA to match clusters to a day assignment (Fig. 1b).

### RNA extraction, library preparation, and sequencing

We collected RNA from iPSCs (day 0) before adding day 1 media and then every 24 h during the differentiation time course for a total of four time points representing intermediate cell populations from iPSCs to definitive endoderm (Additional file 2: Figure S1). We extracted the RNA using the ZR-Duet DNA/RNA MiniPrep kit (Zymo) with the addition of an on column DNAse I treatment step before RNA elution. We used non-strand-specific, polyA capture to generate RNA-seq libraries according to the Illumina TruSeq protocol. To estimate the RNA concentration and quality, we used the Agilent 2100 Bioanalyzer (Additional file 1: Table S1; Additional file 2: Figure S1). We added barcoded adaptors (Illumina TruSeq RNA Sample Preparation Kit v2) and sequenced the 50 bp single-end RNA-seq libraries on the Illumina HiSeq 4000 at the Functional Genomics Core at University of Chicago on two flowcells (Additional file 1: Table S2). To minimize the introduction of biases due to batch processing, we chose the RNA extraction batches, library preparation batches, sequencing pools, adaptor names, and flowcells in a manner that maximally partitioned the biological variables of interest (day, species, cell line; Additional file 1: Tables S2A and S4).

We generated a minimum of 14,424,520 raw reads per sample. We used FastQC (http://www.bioinformatics.babraham.ac.uk/projects/fastqc/) to confirm that the reads were high quality.

### Quantifying the number of RNA-seq reads from orthologous genes

We mapped human reads to the hg19 genome and chimpanzee reads to panTro3 using TopHat2 (version 2.0.11) [14], allowing for up to two mismatches in each read. We kept on only reads that mapped uniquely. To prevent biases in expression level estimates due to differences in messenger RNA transcript size and the relatively poor annotation of the chimpanzee genome, we only kept reads that mapped to a list of orthologous metaexons across 30,030 Ensembl genes available for the hg19 and panTro3 genomes as described previously [6]. Gene expression levels were quantified using the *featureCounts* function in SubRead 1.4.4 [42]. For one sample (C2B at Day 0), the number of raw reads was approximately double the second highest number of raw reads. Therefore, we subsampled the raw reads to approximately the same number of raw reads as the second highest sample.

We performed all downstream processing and analysis steps in R (version 3.2.2) unless otherwise stated.

### Transformation and normalization of RNA-seq reads

After receiving the raw gene counts, we calculated the log_2_-transformed CPM for each sample using edgeR [43]. To filter for the lowly expressed genes, we kept only genes with an expression level of log_2_(CPM) > 1.5 in at least 16 samples per species [44]. For the remaining genes, we normalized the original read counts using the weighted trimmed mean of M-values algorithm (TMM) [44] to account for differences in the read counts at the extremes of the distribution and calculated the TMM-normalized log_2_-transformed CPM.

When we performed PCA using the TMM-normalized log_2_-transformed log2(CPM) values, we found one outlier (H1B at Day 0, Additional file 2: Figure S2A). We removed this sample from the list of original gene counts. We filtered for the lowly expressed genes by retaining genes with an expression level of log_2_(CPM) > 1.5 in at least 15 human samples and at least 16 chimpanzee samples. In total, 10,304 genes remained. We performed TMM-normalization and then performed a cyclic loess normalization with the function normalizeCyclicLoess from the R/Bioconductor package limma [45, 46]. We found that the TMM-normalized log_2_(CPM) values were highly correlated with the TMM- and cyclic loess-normalized log_2_(CPM) values (*r* > 0.99 in the 63 samples; Additional file 2: Figure S2B). We used the TMM- and cyclic loess-normalized log_2_(CPM) expression values in all downstream analysis unless otherwise stated.

We calculated normalized log_2_-transformed RPKM values by using the function rpkm with normalized library sizes from the package edgeR [43] (Additional file 2: Figure S2C). We measured the “gene lengths” as the sum of the lengths of the orthologous exons and were also used in [12]. This method of calculating RPKM was highly correlated with a method in which we subtracted log_2_(gene length in kbp) from the TMM- and cyclic loess-normalized log_2_(CPM) values (*r* > 0.97).

### Data quality and analysis of technical factors

To assess the data quality, we performed PCA on the normalized log_2_(CPM) values from above (Fig. 2a). PC1 was highly associated with day and PC2 was highly associated with species (*r* > 0.92 for each, Fig. 2a; Additional file 1: Table S4A and B). We sought to determine if the study’s biological variables of interest were confounded with any of the study’s recorded technical aspects (Additional file 1: Table S4C and D). First, we calculated which of our 35 recorded technical factors were statistically significant predictors of PCs 1–5 with individual linear models for each technical factor. The 19 statistically significant predictors (FDR cutoff of 10% assessed on the 5 × 35 matrix) were carried to the second stage. In this stage, we determined which technical factors were associated specifically with either day or species, with individual linear models for each technical factor. We quantified these associations using the *P* values from analysis of variance (ANOVA) for the numerical technical factors and from Chi-squared test (using Monte Carlo simulated *P* values) for the categorical technical factors. Statistical significance was determined by Benjamini–Hochberg adjusted *P* value < 10% (assessed on the 2 × 19 matrix). A variable for cell line includes a species but not a day component and was tested in this pipeline. They were found to each be confounded with species (Benjamini–Hochberg adjusted *P* value < 10^−4^) but not day (Benjamini–Hochberg adjusted *P* value > 0.9), thereby increasing the confidence in our pipeline.

We note that when all sequencing pools (mastermixes) were considered together, there was a relationship between adaptor sequence and day (χ^2^ test, Benjamini–Hochberg adjusted *P* = 0.01); however, this relationship is substantially weaker when “adaptor sequence” and “day” were tested in each of the four sequencing pools separately (Benjamini–Hochberg adjusted *P* > 0.9 in each test). Our most highly dependent variables with day or species were related to properties inherent to the iPSC model, including harvest density and day (Benjamini–Hochberg adjusted *P* = 0.02), harvest density and species (Benjamini–Hochberg adjusted *P* = 0.03), and harvest time and day (Benjamini–Hochberg adjusted *P* = 0.01) (Additional file 1: Table S4D; Additional file 2: Figure S5).

During this analysis, we observed that the purity estimates were relatively similar across days and between species until the final day (Fig. 1b; Additional file 1: Table S1; Additional file 2: Figure S3C). Therefore, it was important to explore how the variance for a given technical factor was partitioned across the biological variables of interest (e.g. across the days, species, and day-by-species interactions). For each recorded technical variable, we created a reduced model and a full model. The reduced model contained only species and day as fixed effects and the technical factor as the response variable. The full model had the same response variable but contained species, day, and a species-by-day interaction as fixed effects. We then compared the two models and reported the significance (Additional file 1: Table S11).

The exact tools used to compare the two models were data-dependent (Additional file 1: Table S2 and columns 1–2 in Table S11). For numerical data (24 technical factors), we constructed the full and reduced normal general linear models for each technical factor. We compared the models using ANOVA and extracted the *P* value directly from ANOVA. For categorical data with two levels (three technical factors), we constructed the two general linear models from the binomial family. We used ANOVA to compare the models and extracted the deviance along with its degrees of freedom. Based on the deviance, we calculated the Chi-squared statistic and associated *P* value. Eight technical factors (such as RNA extraction data) contained categorical data with more than two levels. We modeled this data type with multinomial logistic regression with the R/Bioconductor package nnet [47] and used ANOVA to obtain the likelihood ratio statistic and associated *P* value. We performed this process for each technical factor using data from days 0 and 1 as well as from days 0 to 3 (Additional file 1: Table S11).

### A linear model-based framework to perform pairwise differential expression analysis

Differential expression was estimated using a linear model based empirical Bayes method implemented in the R package limma [48, 49]. In order to use a linear modeling approach with RNA-seq read counts, we calculated weights that account for the mean-variance relationship of the count data using the function voom from the limma package [50]. This limma+voom pipeline has previously been shown to perform well with *n* > 3 biological replicates/condition [51, 52].

For all pairwise differential expression comparisons, the species, day, and a species-by-day interaction were modeled as fixed effects and individual as a random effect. The individual (cell line) rather than differentiation batch was modeled as a random effect because when using a linear model, individual was most highly correlated with PCs 2 and 3, whereas batch was most highly correlated with PC 10. Since our recorded technical factors were not confounded with our biological variables of interest and did not contribute significantly to the first five PCs of variation (Additional file 1: Table S4A–E), we did not include any other covariates.

We used contrast tests in limma to find genes that were DE by species at each day (Additional file 1: Table S5), DE between days for each species (Additional file 1: Table S6), and significant day-by-species interactions for days 1–3 (Additional file 1: Table S8). For each pairwise DE test, we corrected for multiple testing with the Benjamini–Hochberg false discovery rate (FDR) [53] and genes with an FDR-adjusted *P* value < 0.05 were considered DE unless otherwise stated in the text.

To find the number of shared DE genes in consecutive time points in each species (Fig. 4), we used a two *P* value cutoff system. To be “shared” across species for a given pair of time points (e.g. day 0 to 1), a gene must have an FDR-adjusted *P* value < 0.01 in one species and an FDR-adjusted *P*-value < 0.05 in the other species [53]. To estimate the percentage of DE genes in chimpanzees given the observation in humans, we divided the number of genes with an FDR-adjusted *P* value < 0.01 in chimpanzees over the number of genes with an FDR-adjusted *P* value < 0.05 in humans.

### Combining technical replicates

Some analyses did not allow us to model technical replicates explicitly and treating them as biological replicates would introduce bias in the data. Therefore, we combined technical replicates for the same individual, when available. We calculated the average of the normalized log_2_(CPM) values for each cell line at each time point. For day 0, one human cell line had a pair of technical replicates that were averaged together. For days 1–3, two human cell lines had technical replicates that were averaged. We were able to average technical replicates for each of the four chimpanzee cell lines at each time point. After this process, six human data points and four chimpanzee data points per day remained, for a total of 40 data points.

When we performed PCA using these 40 data points, the results were similar to the PCA plot including all the technical replicates (Additional file 2: Figure S9A); PC1 was still correlated with day and PC2 was correlated with species (Additional file 1: Table S4E). We visually inspected the PCA plot for the distinct clustering of data points with averaged technical replicates and single replicates in the humans, and this potential pattern was not present, increasing our confidence that this process did not introduce bias into the data.

We found that the expression values for the 40 samples were robust with respect to the method used to combine the technical replicates. The post-normalization method described above was strongly correlated with a pre-normalization method to combine technical replicates (*r* > 0.99 for the 10,304 genes included in the main analysis; Additional file 2: Figure S9B). In our pre-normalization method of combining the technical replicates, we summed the raw counts of technical replicates at each time point (for a total 40 data points) and performed the normalization steps described in the “Transformation and normalization of RNA-sequencing reads” section.

### Joint Bayesian analysis with Cormotif

To cluster genes by their temporal gene expression patterns, we used the R/Bioconductor package Cormotif (version 1.22.0), a method that jointly models multiple pairwise differential expression tests [19]. Unlike other available methods in this class, the Cormotif framework allows for dataset-specific differential expression patterns. To identify patterns in expression over time (called “correlation motifs”), expression levels from days 1–3 were compared to those to the previous day for each gene in each species. Since the program does not allow for the explicit modeling of technical replicates (unlike the voom+limma method above), we first ran the program with the expression values averaged across technical replicates. For more information on this process, see the “Methods” section on “Combining technical replicates.”

To use Cormotif, we were required to specify the number of correlation motifs to model. We determined a reasonable range by investigating both the Bayesian information criterion (BIC) and Akaike information criterion (AIC). We observed that the BIC and AIC were minimized across many seeds when seven or eight correlation motifs were modeled, respectively (Additional file 1: Table S15; Additional file 2: Figure S8A). Thus, we further explored models with seven and eight correlation motifs. Because Cormotif is not deterministic, we ran Cormotif 100 times and recorded the seed that produced the model with the largest log likelihood (Additional file 1: Table S15). The best model (the seed with the greatest log likelihood) with seven correlation motifs is displayed in Additional file 2: Figure S8B and the best model with eight correlation motifs is featured in Fig. 5. We selected the model with eight correlation motifs to be the primary figure because it had a large log likelihood and all motifs contained > 100 genes. It should be noted, however, that the two models had very similar correlation motifs (expression patterns; Additional file 4).

We were initially conservative when assigning a gene to a specific correlation motif. Following the advice of the Cormotif authors [19], a gene must have a posterior likelihood estimate of ≥ 0.5 to be called DE between time points and < 0.5 to be considered not DE (Additional file 1: Table S9A). We also used this assignment criteria when using Cormotif to compare expression levels using different combination methods (Additional file 2: Figure S8C) and to compare all time points to day 0 (Additional file 2: Figure S8D). For a trajectory to be defined as DE, the trajectory in humans and chimpanzees needed similar posterior probabilities of differential expression (≤ 0.20) at each comparison along the trajectory.

Using topGO [20], we tested for enrichment of Gene Ontology (GO) biological processes enrichment analysis on various combinations of correlation motifs (Additional file 1: Table S9B–D). To test for significance, we used the same parameters as [54]. This included the use of Fisher’s exact test, with topGO’s weight01 algorithm to account for the correlation among GO categories in its graph structure. Categories with *P* value < 0.01 were considered significant. To determine the categories enriched in the Motif 4 + 7 group only, we ensured that these categories were not significant in any other group (*P* < 0.01; Additional file 1: Table S9B–D). To test this in a more rigorous way, we then compared the enriched categories for the Motif 4 + 7 group given the genes in the two other groups (Additional file 1: Table S9E) using the compareCluster function in the R package clusterProfiler [21]. We used a q value cutoff of 0.05 [53] and set the size of genes annotated by Ontology term for testing parameter between 3 and 3000.

### Global analysis of variation in gene expression levels

We calculated the variance in gene expression level for each gene in each species. Since the largest theoretical range of a variance is from 0 to infinity, we performed a log_2_ transformation to each variance value (Fig. 6). For the analysis with lymphoblastoid cell lines (LCLs) and the four tissues, we used normalized gene expression data from the GTEx Portal (release V6p; gene expression data is in RPKM) from LCLs and four additional tissues [23, 55]. Three of the four tissues are derived from the endoderm germ layer—liver, lung, and pancreas. The heart tissue is mesoderm-derived, for comparison. We identified all of the genes that were expressed and passed GTEx’s filtering criteria (*n* = 17,542 genes) as well as the individuals that had contributed samples to all five tissues (*n* = 6).

We then calculated and plotted the log_2_ variance of the normalized gene expression using the same pipeline as before (Additional file 2: Figure S10B). For samples with associated purity values (*n* = 30), we calculated the effect of purity gene expression levels. We regressed out effect of purity on gene expression levels on a gene-by-gene basis using the linear model function in R in each species independently. We then calculated the log_2_ variance of these residuals for each gene. We shifted the log_2_ variances for these residuals so that the median at day 0 for each species = 1. For samples without purity values (*n* = 32), we calculated the log_2_ variance of the gene expression levels for each gene and scaled the values so that the median at day 0 for each species = 1. For each gene at each day for each species, we averaged the scaled log_2_ variances of the residuals and the log_2_ variances of the gene expression levels (Additional file 2: Figure S10B). We then performed a one-sided t-test between the distribution of log_2_(variances in gene expression) from day 0 and day 1 in each species, with the alternative hypothesis that the variation was greater in day 0 than day 1.

We compared the effect sizes of interspecies DE genes with a one-sided Mann–Whitney *U* test on magnitudes of effect sizes (Additional file 2: Figure S10C). We tested the null that there was no change in log_2_ fold change in gene expression across the species from day 0 to day 1, with the alternative hypothesis that the average magnitude of effect of DE genes (FDR = 5%) was greater in day 0 than day 1.

### Gene-by-gene analysis of variation in gene expression levels and calculating the proportion of true positives

To determine if there was an enrichment of genes undergoing changes in variation one species, we used an F test to compare two variances in R (var.test command) for each gene using the averaged log_2_(CPM) expression values of technical replicates. In these tests, the null hypothesis was no change of variance in the gene expression levels between days and the alternative hypothesis was a reduction in variation of gene expression levels between two time points (a one-sided test).

We calculated the *P* values for the F statistics from each test and plotted the densities using ggplot2 [56]. If a *P* value distribution appeared to be even slightly skewed towards small *P* values, we used the R package qvalue to determine $\widehat{\pi}$_o_, the true proportion of null statistics from a given *P* value distribution [24]. Its complement, $\widehat{\pi}$_1_, is considered the proportion of significant tests from a *P* value distribution. We used this process to analyze the reduction in variation in each species from days 0 to 1, 1 to 2, 2 to 3, and 1 to 3 (Fig. 7a and b).

Afterwards, we used the same procedure (F tests) to test the alternative hypothesis that the variation of gene expression increased between two time points. We determined $\widehat{\pi}$_o_ and $\widehat{\pi}$_1_ in the same manner as above to analyze the increase in variation in each species from days 0 to 1, 1 to 2, 2 to 3, and 1 to 3 (Fig. 7e–f).

### Estimating the proportion of genes that undergo a change in variation in both species

We then estimated the true proportion of significant genes shared across species for a given set of time points. Rather than take the intersection of the significant genes (for which we would be underpowered), we adopted a method from Storey and Tibshirani 2003 (Storey’s $\widehat{\pi}$_o_) [24]. This method was recently implemented by Banovich et al. to determine the sharing of quantitative trait loci (QTLs) from different cell types [22, 24]. Using the *P* value distributions generated in the previous section, we subset the genes in species 2 conditioned on its F statistic significance in species 1 (unadjusted *P* value < 0.05). To test for an enrichment of small *P* values, we used the *P* values from species 2 to determine $\widehat{\pi}$_o_ using the same process as the previous section (Figs. 7c–f, Additional file 2: Figure S13). We then repeated this process for other *P* value cutoffs, including 0.01 and 0.10 (Additional file 2: Figures S15 and S16). To determine robustness with respect to the number of genes considered significant in species 1, we calculated $\widehat{\pi}$_o_ for species 2 conditioned on 100 genes with the lowest *P* values in species 1. This process was repeated for the top 101 to all 10,304 genes (Additional file 2: Figure S14).

### Estimating the null hypothesis for the proportion of genes that undergo a change in variation in both species

To determine the null hypothesis for the $\widehat{\pi}$_1_ based on conditioning, we performed permutation tests. First, we combined the unadjusted *P* values from the F test for a reduction in variation from days 0 to 1 in chimpanzees (species 1) and humans (species 2). We used the randomizeMatrix function in the R package picante [57] to permute the *P* values of species 1 and then merged this *P* value distribution with the *P* value distribution from species 2. We then determined $\widehat{\pi}$_o_ in species 1 conditioned on its *P* value significance in species 2 (unadjusted *P* value < 0.05). We repeated this process a total of 100,000 times and found the complement of the 100,000 values (Additional file 1: Table S13). We defined the permuted null hypothesis as the mean $\widehat{\pi}$_1_ value. We then repeated this process, with humans as species 1 and chimpanzees as species 2.

For the enrichment analysis, we classified the genes with a reduced variation of gene expression in both species using a 2 *P* value cutoff in the results of the F tests (*P* < 0.05 in one species and *P* < 0.10 in the other species). We chose this method because names of the overlapping genes cannot be determined by Storey’s approach [24]. As described in the “Methods” section, “Joint Bayesian Analysis with Cormotif,” we performed the enrichment analysis using topGO with a significance of *P* < 0.01 [20]. To determine the genes associated with embryonic lethality response to perturbation, we entered our list of genes into the Mouse Genome Database at the Mouse Genome Informatics website provided by the Jackson Laboratory (http://www.informatics.jax.org/batch, [26, 27, 58, 59]) and selected the “Mammalian Phenotype” option [28]. With this output [28], we eliminated any feature type that was not a protein-coding gene and those genes without an associated phenotype. For each gene list, we calculated how many genes contained at least one of the following terms: “embryonic lethality”, “prenatal lethality,” or “lethality throughout fetal growth and development.”

## Additional files

Additional file 1: Additional tables. (XLSX 17200 kb)

Additional file 2: Additional figures. (PDF 10000 kb)

Additional file 3: TMM- and cyclic loess-normalized log_2_ counts per million for the 10,304 genes analyzed in this study for the 63 samples used in the downstream analysis. Each row is an Ensembl gene name and each column is a Sample ID. (RDA 4700 kb)

Additional file 4: Additional text and methods. Provides additional information about the Joint Bayesian analysis and a correlation-based method. (DOCX 32 kb)
