# Supplementary material for: A comparative study of endoderm differentiation in humans and chimpanzees
Source: Genome Biol. 2018 Oct 15;19:162. doi: 10.1186/s13059-018-1490-5 (PMC6191992; doi:10.1186/s13059-018-1490-5)
Supplement: Supplementary file 2 — Additional figures. (PDF 10000 kb) [file 13059_2018_1490_MOESM2_ESM.pdf]

S1.

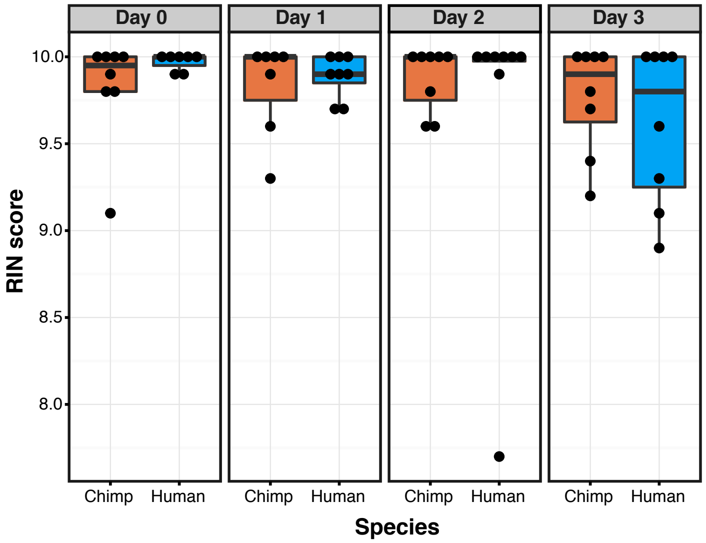

Figure S1: RNA Integrity Number (RIN) scores across biological variables of interest. RNA quality was not confounded with day, species, or batch (Benjamini-Hochberg adjusted P value > 0.10 for individual linear models). Each point represents one sample.

S2A. **S2B.** **S2C.**

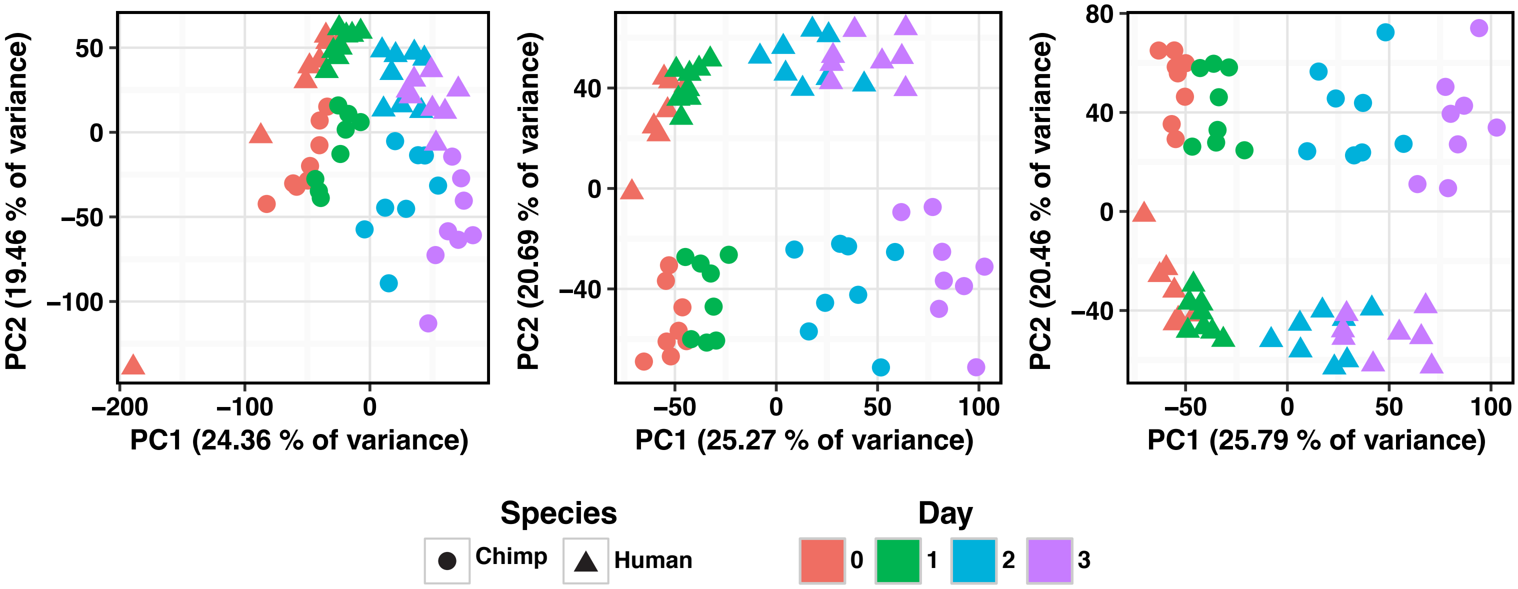

Figure S2: Principal components analysis (PCA) of normalized data. A. Prior to the removal of one outlier sample (H1B at day 0). PC1 separates the data from H1B at day 0 from all other samples. **B.** PCA of the TMM-normalized log2-transformed CPM. PC1 separates the samples by time point (day). PC2 separates the human and chimpanzee samples. **C.** PCA of the library-size normalized log2 reads per kilobase of transcription per million mapped reads (RPKM). PC1 is strongly correlated with cell state (day). PC2 is strongly associated with species.

S3A.

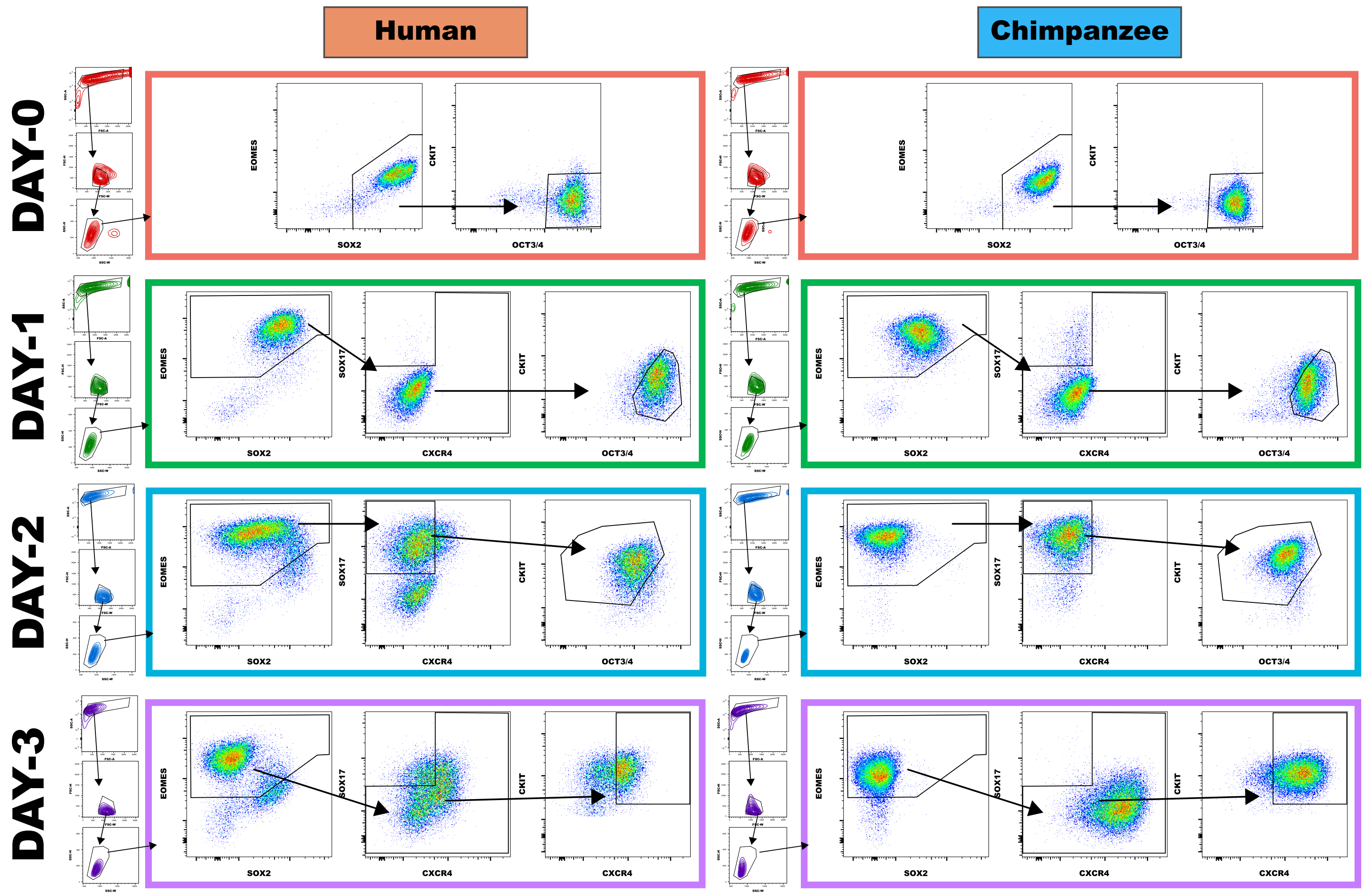

S3B.

Day	Marker					
	SOX2	OCT3/4	EOMES	SOX17	CKIT	CXCR4
0	+ High	+ High				
1	Low	+ High	+		Low	
2		Low	+	+	+ High	
3			+		+	+

S3C.

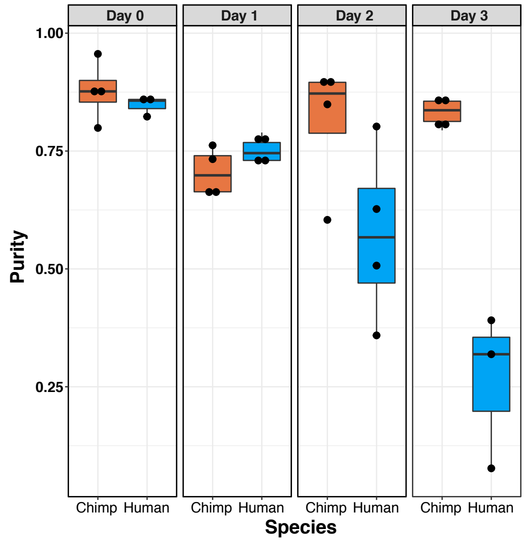

Figure S3: Purity gating information. A. Gating example for human (H5B) and chimpanzee (C4B) timecourses. Dead cells, debris and doublets were removed prior to analysis of fluorescent markers using SSC and FSC gates as shown to the left of each panel.
B. Summary of purity gating scheme. Minimal florescence is expected for the markers in black.
C. Box plot of purity estimates by day and species.

S4.

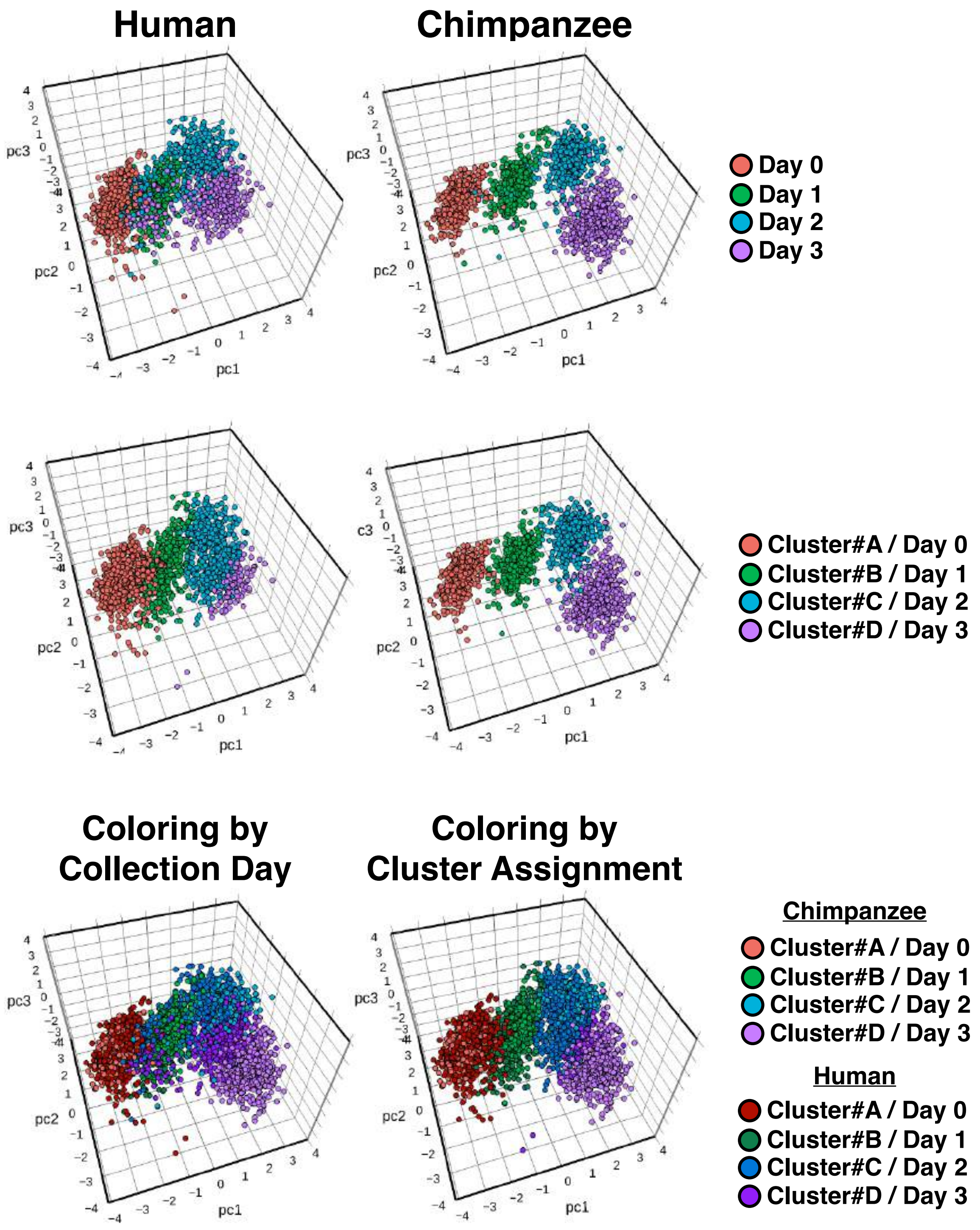

Figure S4: Clustering of fluorescence values. Compensated fluorescence values projected onto the axes of the first three principal components. For plotting, we randomly sampled 400 cells from each individual (total of 1600 cells per species) from each day separately in humans (left panel) and chimpanzee (right panel). Thus each plot is a composite of all days and all individuals, for a given species. The same set of 1600 cells is used throughout the three panels and only the coloring indicating harvest day or cluster assignment is changed.

S5A.

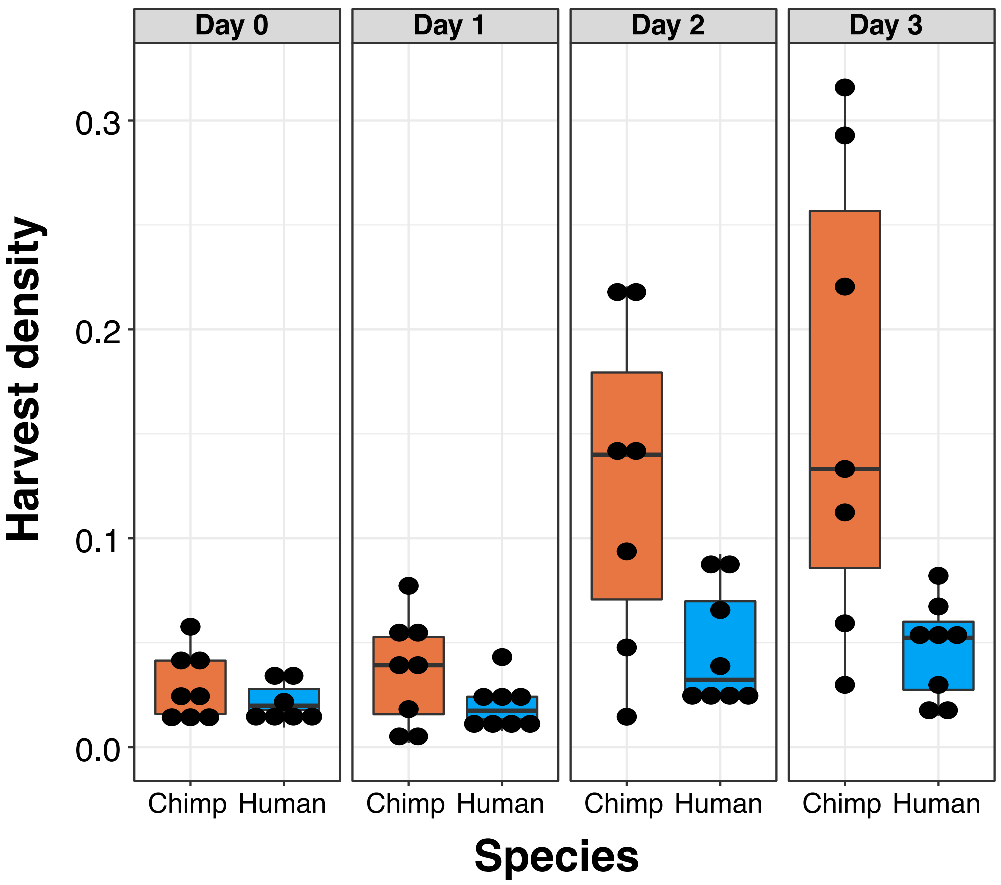

S5B.

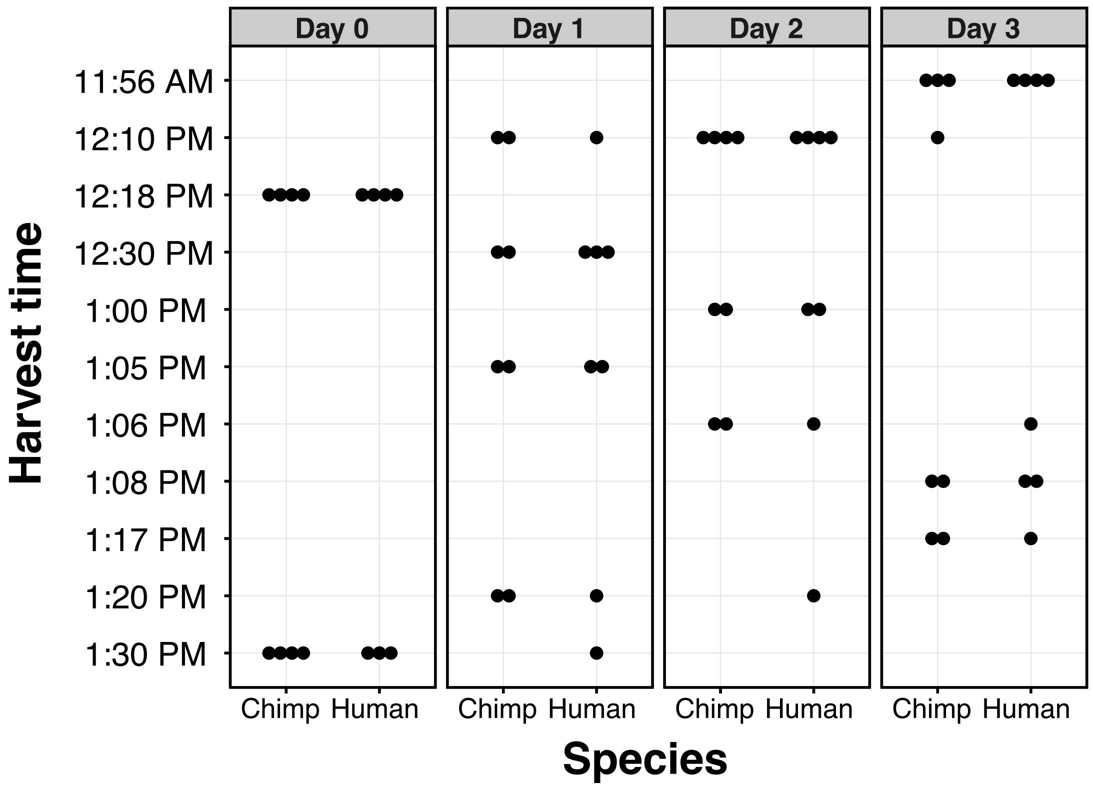

Figure S5: Distribution of potential confounder variables by day and species. A. Harvest densities for each sample by day and species. **B.** Harvest times for each sample by day and species.

S6A.

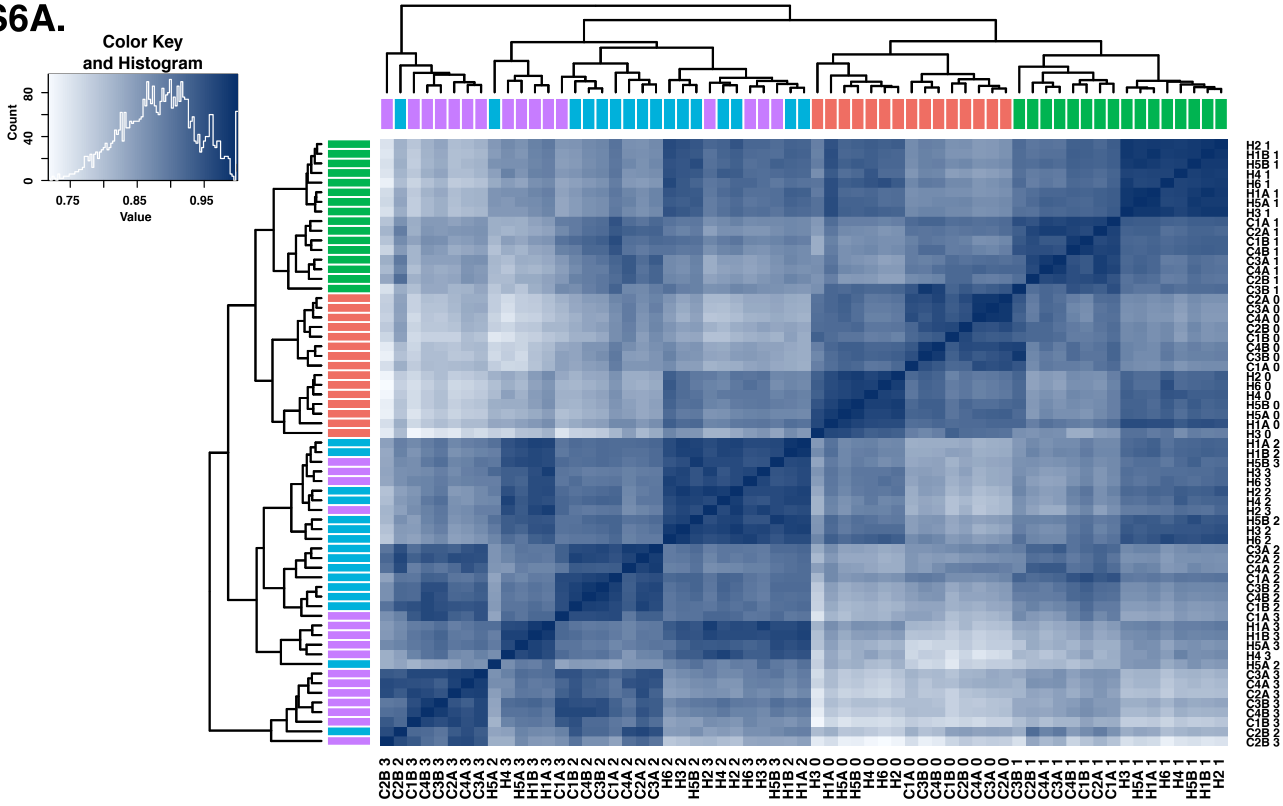

S6B.

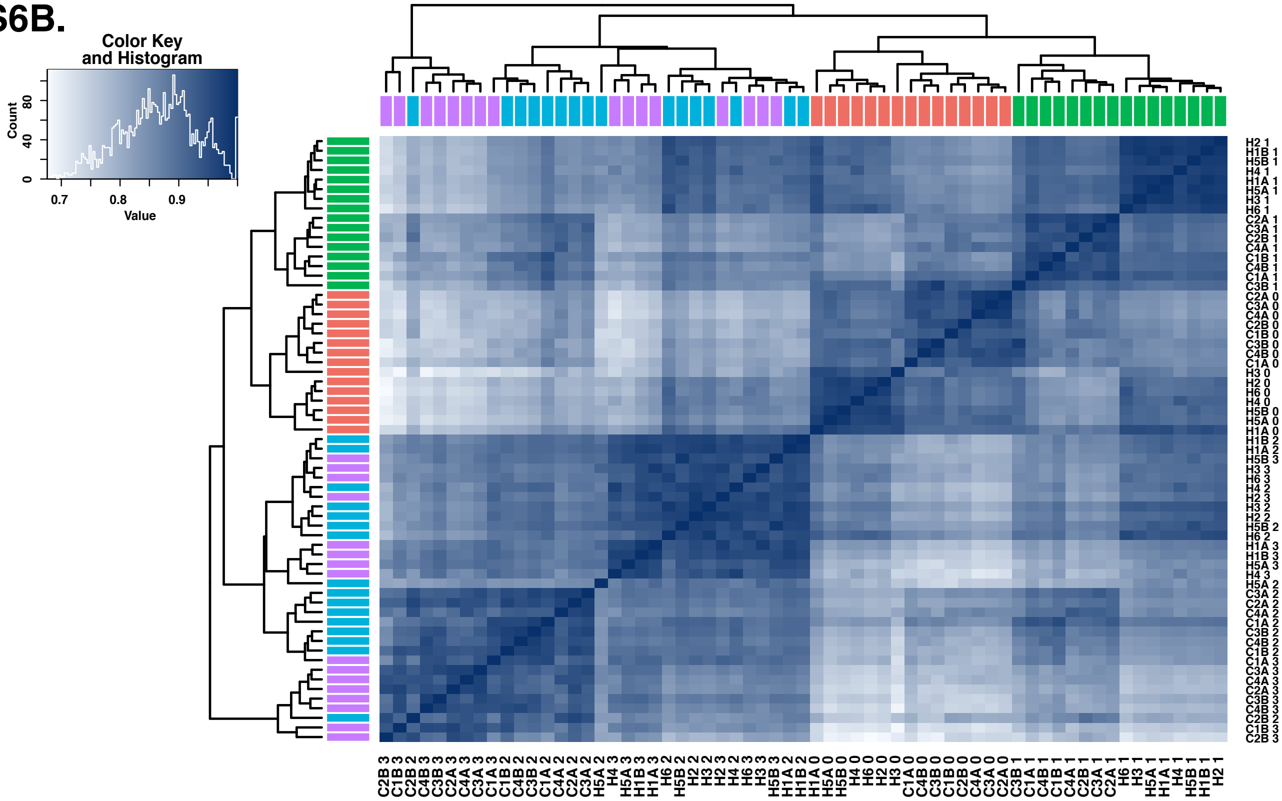

Figure S6: Correlation matrices of normalized $\log_2$(CPM) gene expression values from 10,304 genes. A. Heat map of the Spearman's correlation matrix of normalized $\log_2$(CPM) gene expression values from 10,304 genes. Each square represents the Spearman's correlation of the normalized expression values between two samples. Samples from the same day tend to be more highly correlated (dark blue) than samples from different differentiation days (light blue). **B.** Heat map of the Pearson's correlation matrix of normalized gene expression values from 10,304 genes. The results are similar to (**A**).

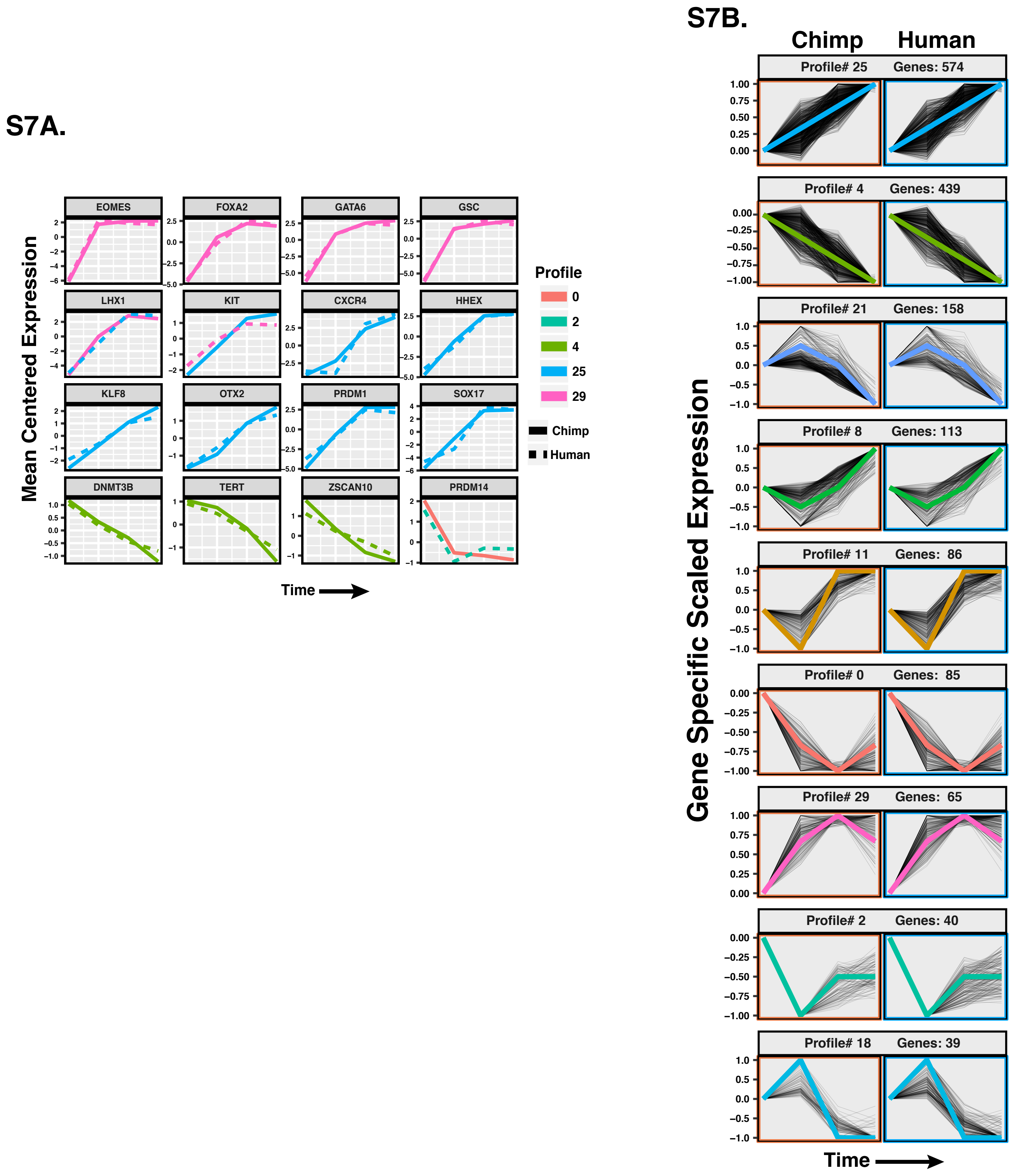

Figure S7: Classifying genes into temporal profiles with Short time course expression miner (STEM) based on TMM-normalized $\log_2$(CPM) expression values. A. Temporal profiles for 16 known critical regulators or markers of the differentiation process. Chimpanzee time course is shown using a solid line while the human time course is shown using a dashed line. Expression is averaged and scaled by mean centering in order to demonstrate magnitude differences at higher resolution. **B.** Temporal profiles determined significant for either chimpanzee or human using STEM. Only genes that were assigned to the same cluster in both species were plotted (grey lines). The colored lines indicate the canonical model profile as defined by STEM. Colors indicate cluster assignment and are the same as used in (**A**). Expression across the time course is averaged and scaled (constraining the data to fall between 1 and -1 by dividing by the absolute value of the most extreme value observed throughout the time course) for each gene.

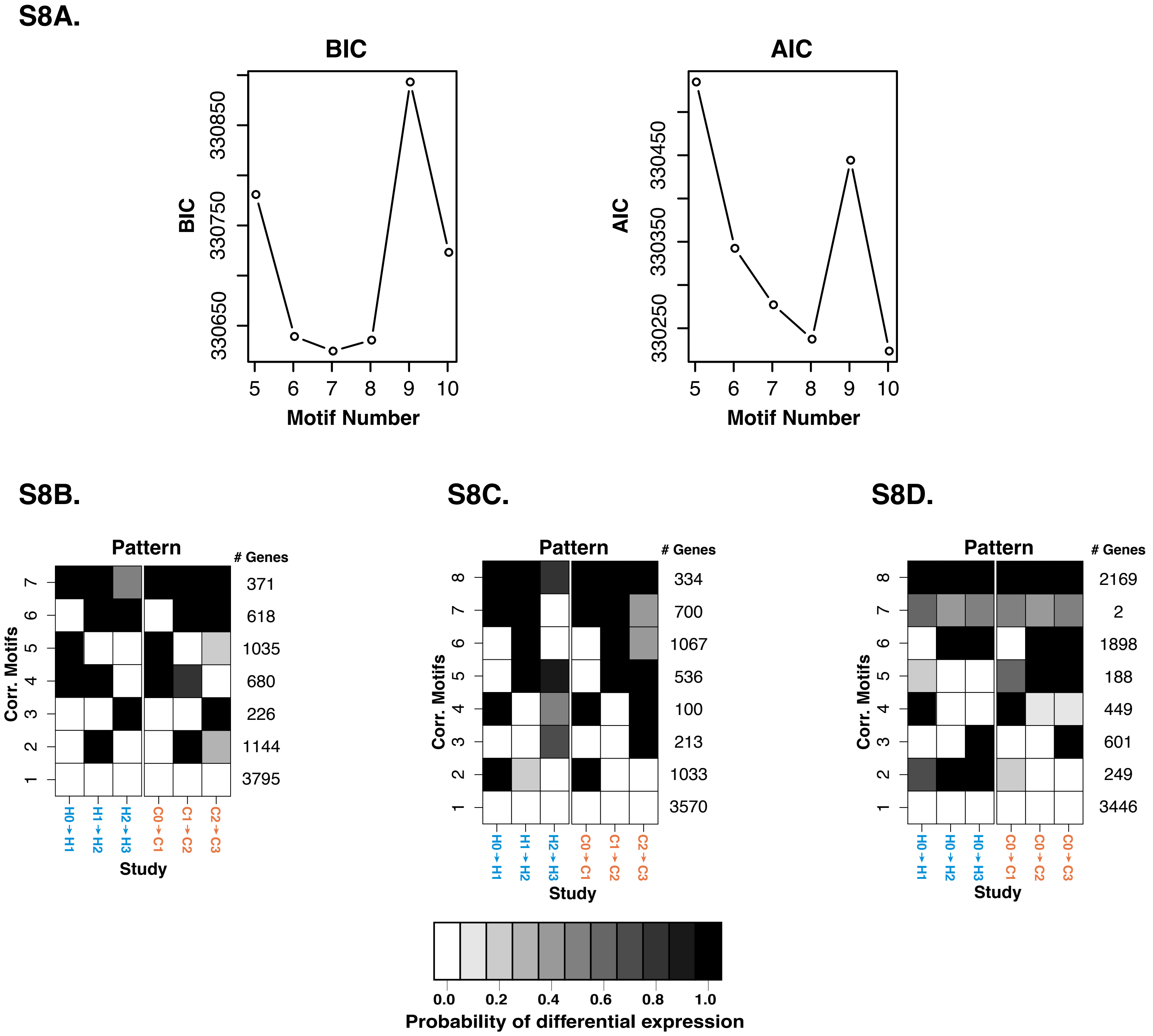

Figure S8: Assessing the robustness of Cormotif results. The shading of each box represents the posterior probability that a gene is DE between two time points in a given species. Each row ("correlation motif") represents the most prevalent expression patterns along the trajectory. The Bayesian information criterion (BIC) and Akaike information criterion (AIC) are goodness-of-fit measurements for the number of motifs in a model (for a given seed). **A.** The BIC and AIC for models from seed 12345 allowing for 5 to 10 correlation motifs. These plots highlight that the BIC and/or AIC was often minimized in models with 7 and 8 correlation motifs. **B.** The predominant expression patterns from a model with 7 correlation motifs (seed 4, see Methods for information about seed selection). The degree of conservation across species calculated using this model is slightly higher than the estimate from Figure 5 (80%, see Additional File 3 for more information). **C.** The 8 correlation motifs and number of genes assigned to the correlation motifs when using a pre-normalization method to combine technical replicates (seed 66). The predominant expression patterns and the number of genes assigned to each expression patterns are similar to Figure 5. **D.** The 8 correlation motifs and number of genes assigned to the correlation motifs when the data points are compared to day 0 rather than the previous day (seed 66). Here, we also observed similar expression trajectories across species (particularly motifs 1, 3, 4, 6, 7, 8).

S9A.

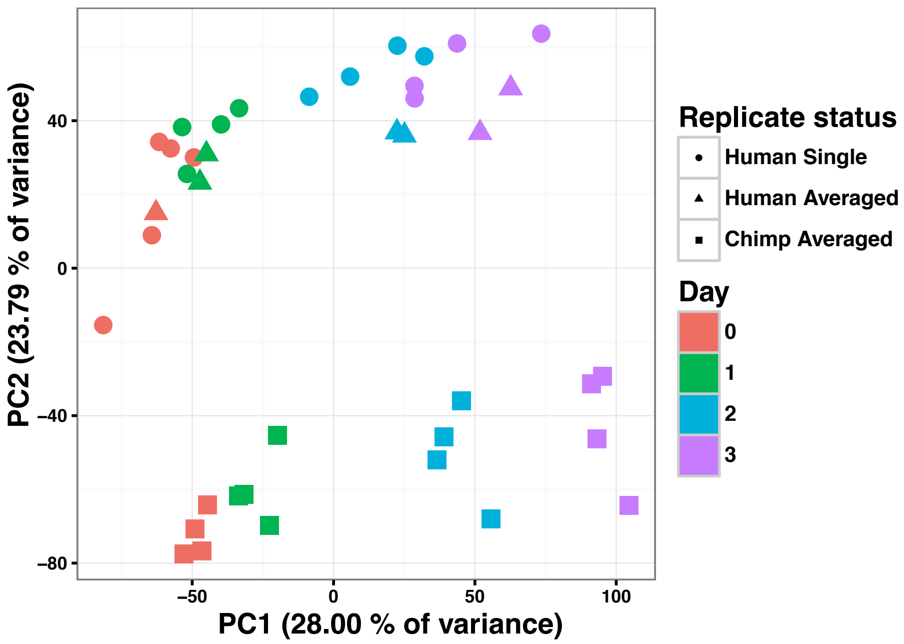

S9B.

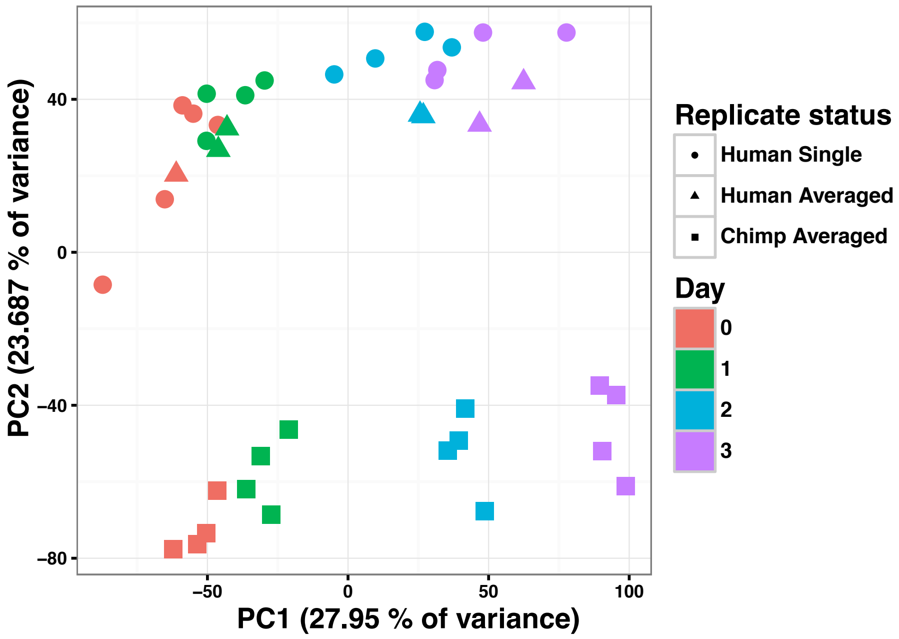

Figure S9: Principal component analysis (PCA) of normalized, combined data (n = 40). A. PCA of the normalized data after averaging the log2(CPM) gene expression values across technical replicates (n = 40 data points). PC1 is highly correlated with time point (day) and PC2 is highly correlated with species. **B.** PCA of the normalized data after combining the gene counts of technical replicates prior to normalization (n = 40 data points). Note that this plot produces similar results to (**A**).

S10A.

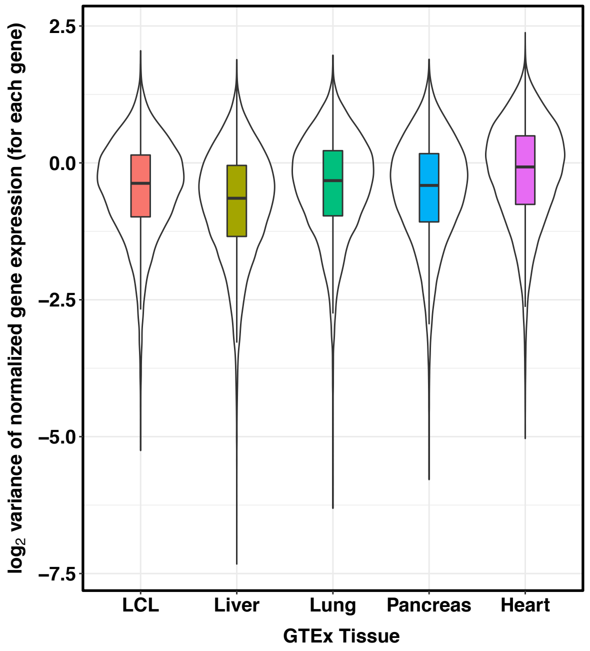

S10B.

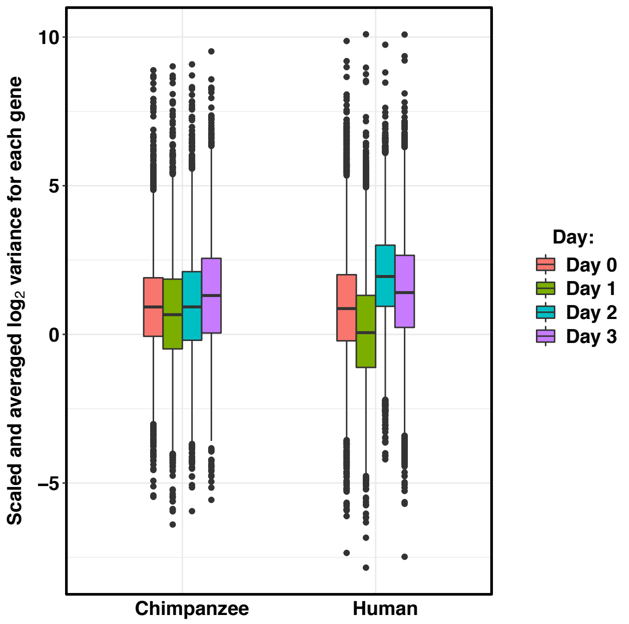

S10C.

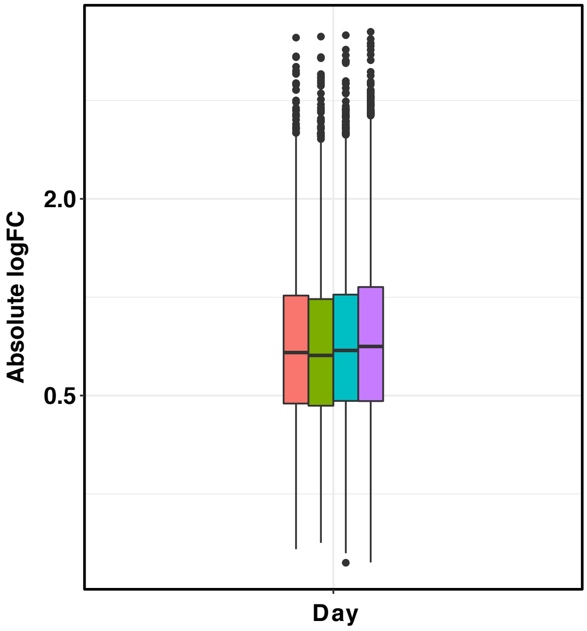

Figure S10: Assessing the variance of gene expression levels. A. Boxplot of the log2 variances of normalized RPKM gene expression levels from lymphoblastoid cell lines (LCLs) and 4 tissues collected by the GTEx Consortium. The distribution of variance of gene expression levels in LCLs are similar to those of the 4 GTEx tissues. **B.** Box plot of the scaled and averaged $\log_2$ variances of gene expression levels for each gene. For samples with associated purity values (n = 30), we regressed out the effect of purity and then calculated the $\log_2$ variance of the residuals for each gene. We shifted the log2 variances of these residuals (n = 30) to match the scales of the $\log_2$ variances of gene expression levels in samples without purity values (n = 32) independently and then averaged the values for each gene. The $\log_2$ variance values are lowest at the primitive streak (day 1). **C.** Distributions of effect sizes of differentially expressed (DE) genes between species across days. Box plot of the absolute value of the log fold change (Absolute logFC) in gene expression between species, at each day. The effect sizes are lowest at the primitive streak (day 1), though the overall effect size is quite modest.

S11A.

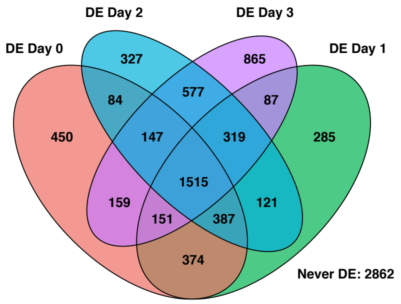

S11B.

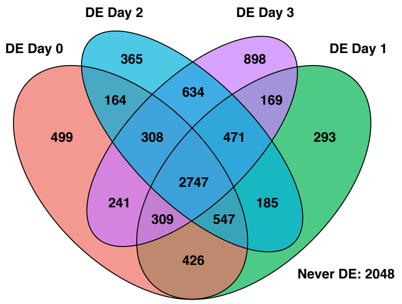

S11C.

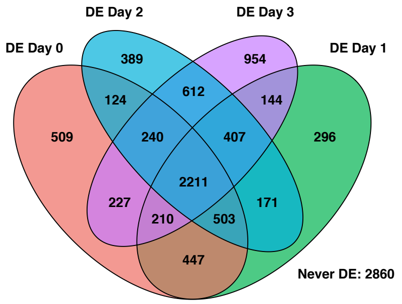

S11D.

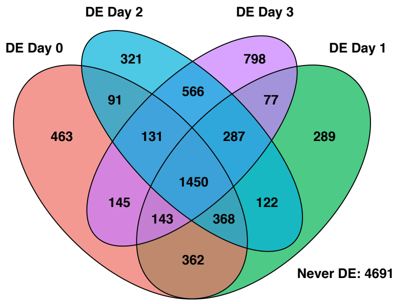

S11E.

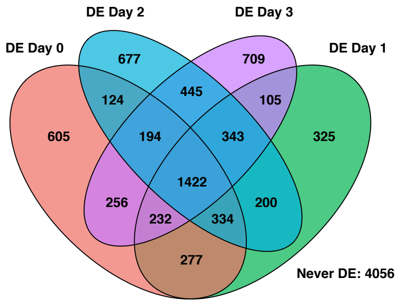

S11F.

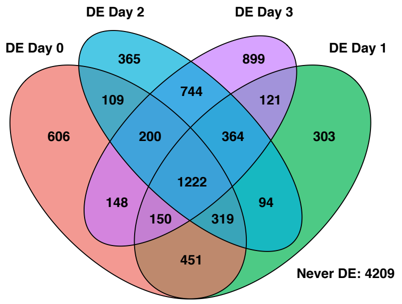

S11G.

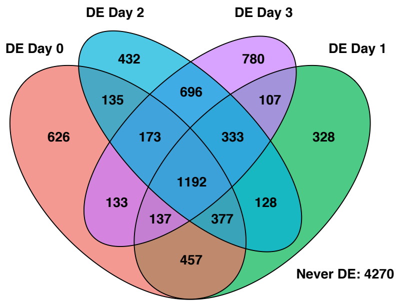

S11H.

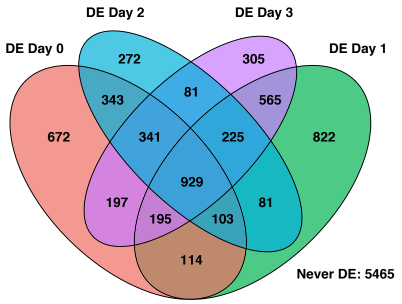

S11I.

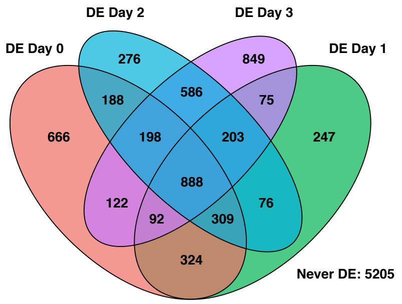

Figure S11: The number of interspecies DE genes using a pairwise, linear model-based approach (limma) is lowest in the primitive streak state. Venn diagram of all DE genes across species at 1% FDR (**A**), 10% FDR (**B**), 5% FDR using normalized RPKM values (**C**), 5% FDR with the samples from differentiation batch 1 (n = 31, **D**), 5% FDR with the samples from differentiation batch 2 (n = 32, **E**), and 5% FDR with only the samples for which we determined purity (n = 30, **F**). **G.** Venn diagram of all DE genes across species at 5% FDR with 24 samples. Each comparison group contained the samples with the 3 highest purity estimates pairs (e.g. 3 human samples at day 0, 3 chimpanzee samples at day 0, etc.). **H.** Venn diagram of all DE genes across species at 5% FDR with 24 samples, using the 3 lowest purity estimates for each of the 8 day-species pairs. **I.** Venn diagram of all DE genes across species with a global correction at 5% FDR. This global correction approach combines the P values from all contrasts into one vector (our study contained 13 total contrasts) and then applies a P value correction on this single vector.

S12A.

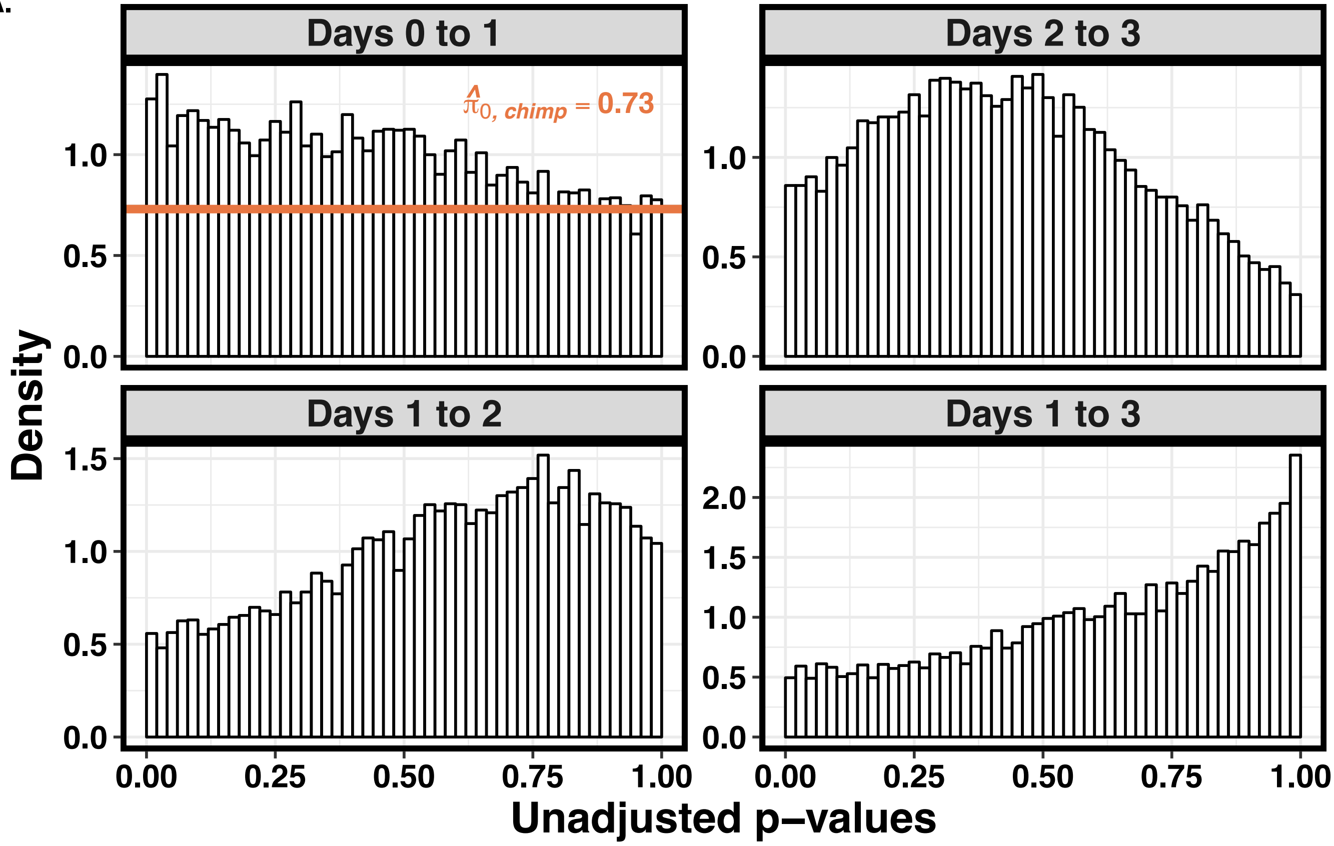

S12B.

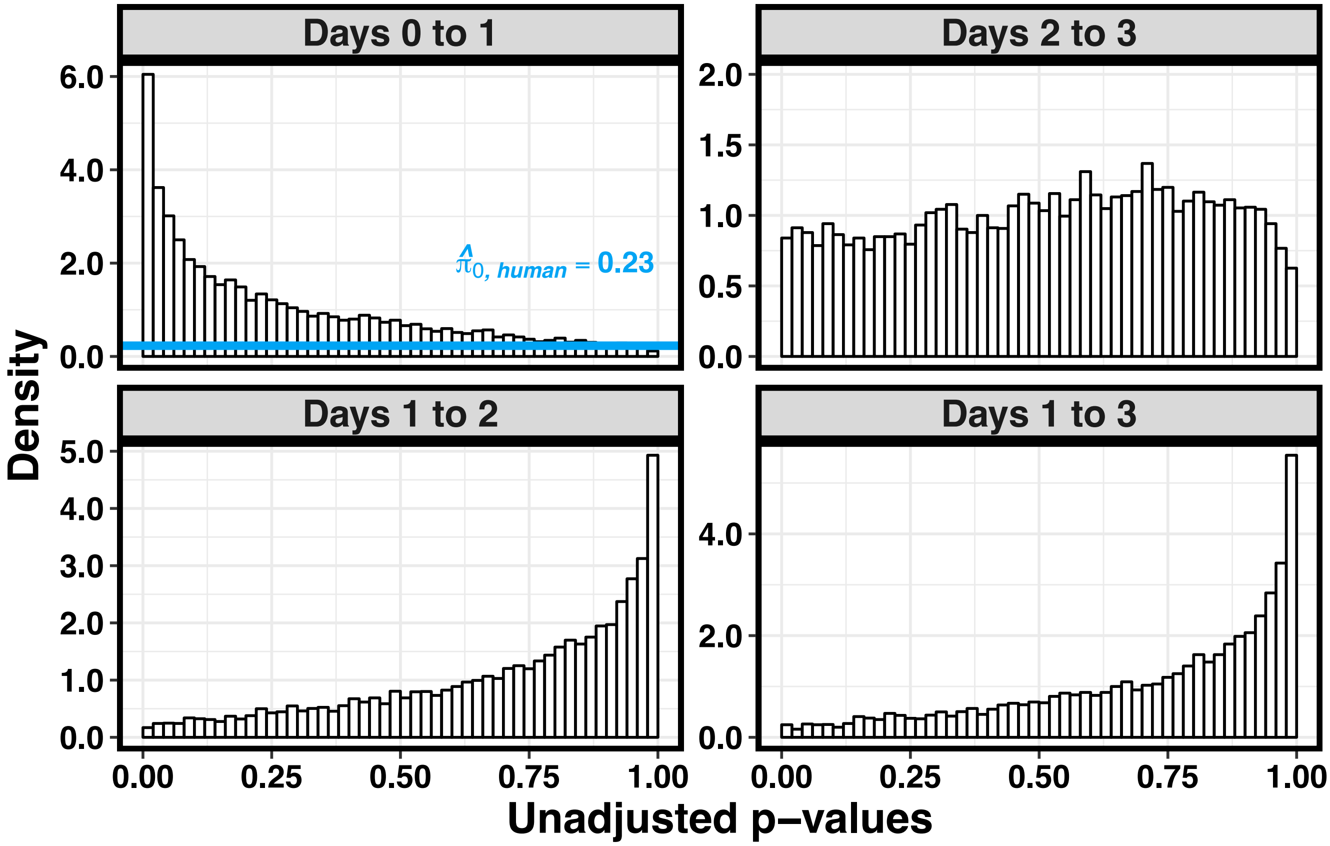

Figure S12: Reduced variation in gene expression at the primitive streak is maintained when using a botstrap method to calculate $\hat{\pi}_0$. results from F tests against the null hypothesis that there was no reduction in variation in gene expression levels (day 1 versus 0, 2 versus 1, 3 versus 2, and 3 versus 1) in the chimpanzee **(A)** and human **(B)** samples. In both plots, the largest proportion of genes with reduced variation occurred at the primitive streak state

S13A.

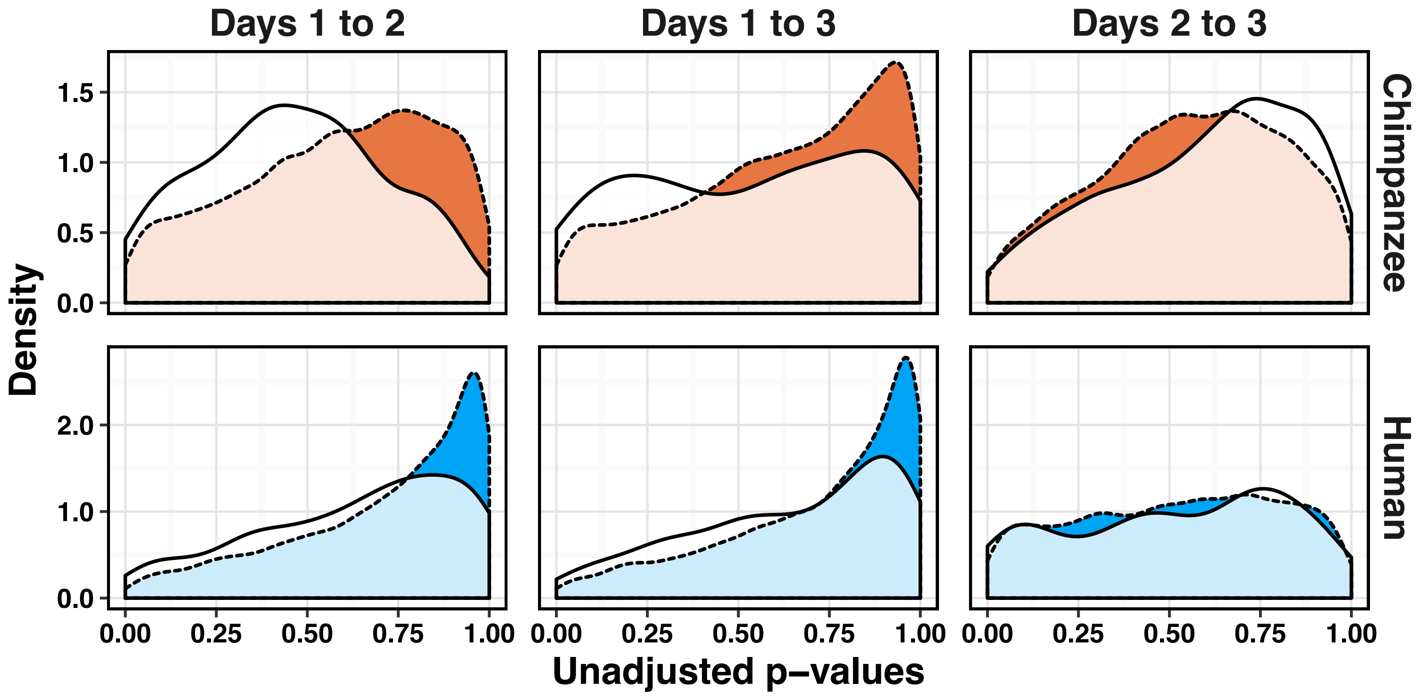

S13B.

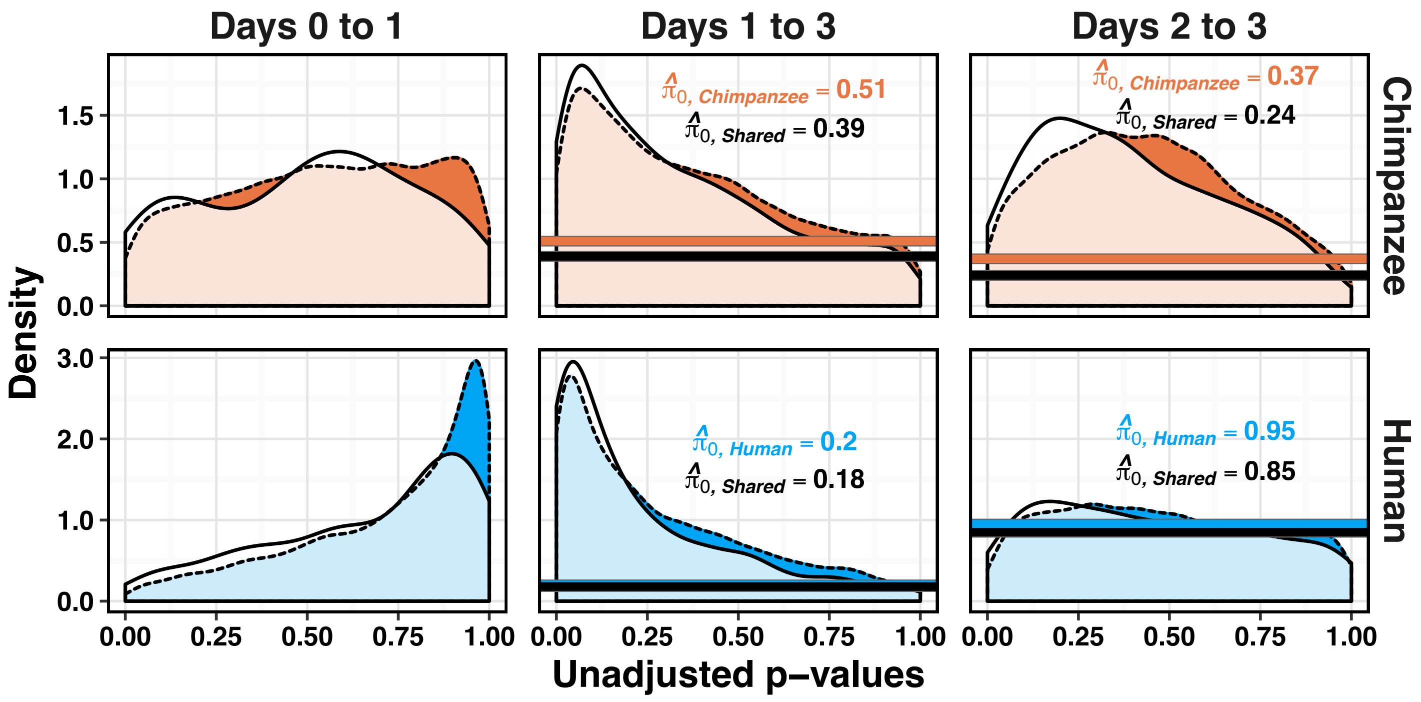

Figure S13: The reduction of variation in gene expression is localized to the primitive streak. $\hat{\pi}_0$ is the estimated proportion of null tests in each distribution. **A.** P value distributions from F tests against the null hypothesis that there was no reduction in variation in gene expression levels as the samples progress along the time course. Each plot shows the P value distribution from F tests for all genes from a given species (orange for chimpanzee and blue for human) and from only genes in that species for which reduced variation was detected in the other species ($P < 0.05$, white). **B.** Same as (**A**) except the results from F tests against the null hypothesis that there was no increase in gene expression levels (increased in day 1 compared to 0, 3 compared to 1, 3 compared to 2).

S14A.

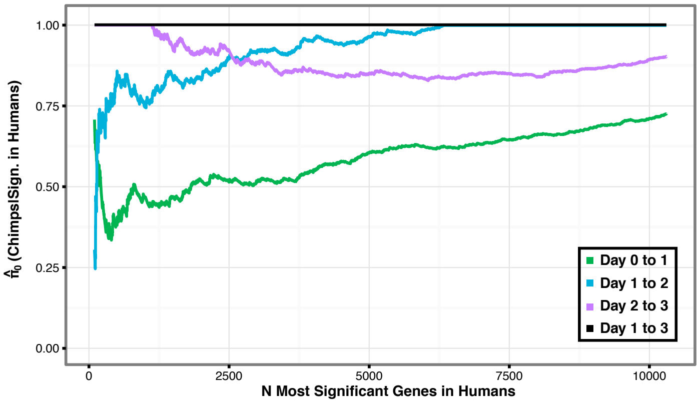

S14B.

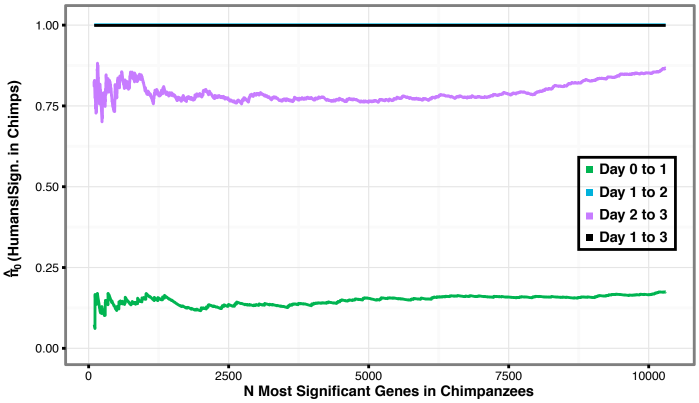

S14C.

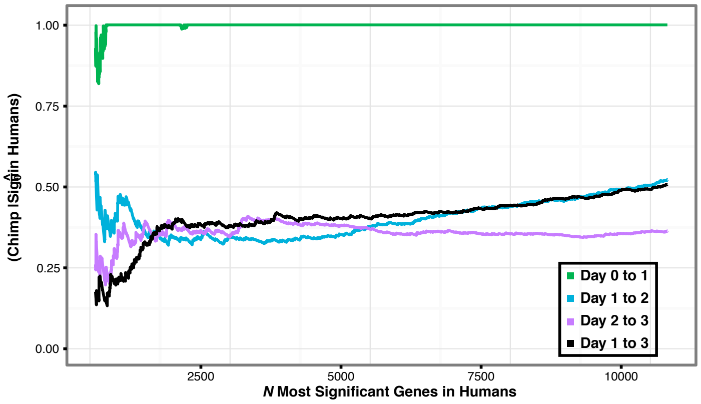

S14D.

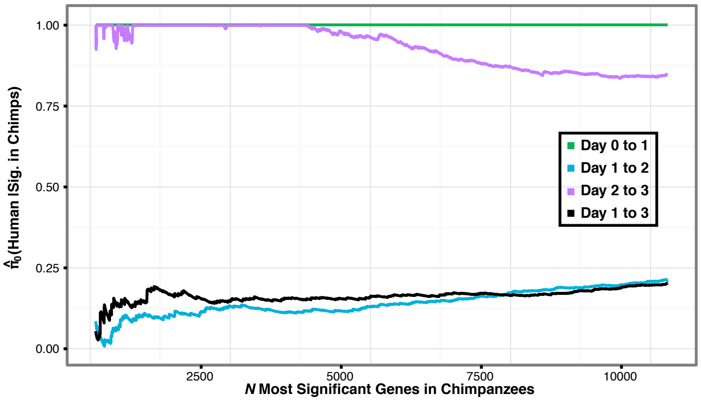

Figure S14: The patterns of change of variation in gene expression are robust with respect to a cutoff based on the number of genes. P value distributions of F tests against the null hypothesis that there was no reduction in variation in gene expression levels as the samples progress along the time course in human **(A)** and chimpanzee **(B)** samples. In each test, Only the N genes whose variation was between states in other species were included. $\hat{\pi}_0$ is the estimated proportion of null tests in each distribution. We then plotted the P value distributions of F tests against the null hypothesis that there was no increase in variation in gene expression levels as the samples progress along the time course in human **(C)** and chimpanzee **(D)** samples. Again, only the N genes whose variation was classified as reduced between states in other species were included.

S15A.

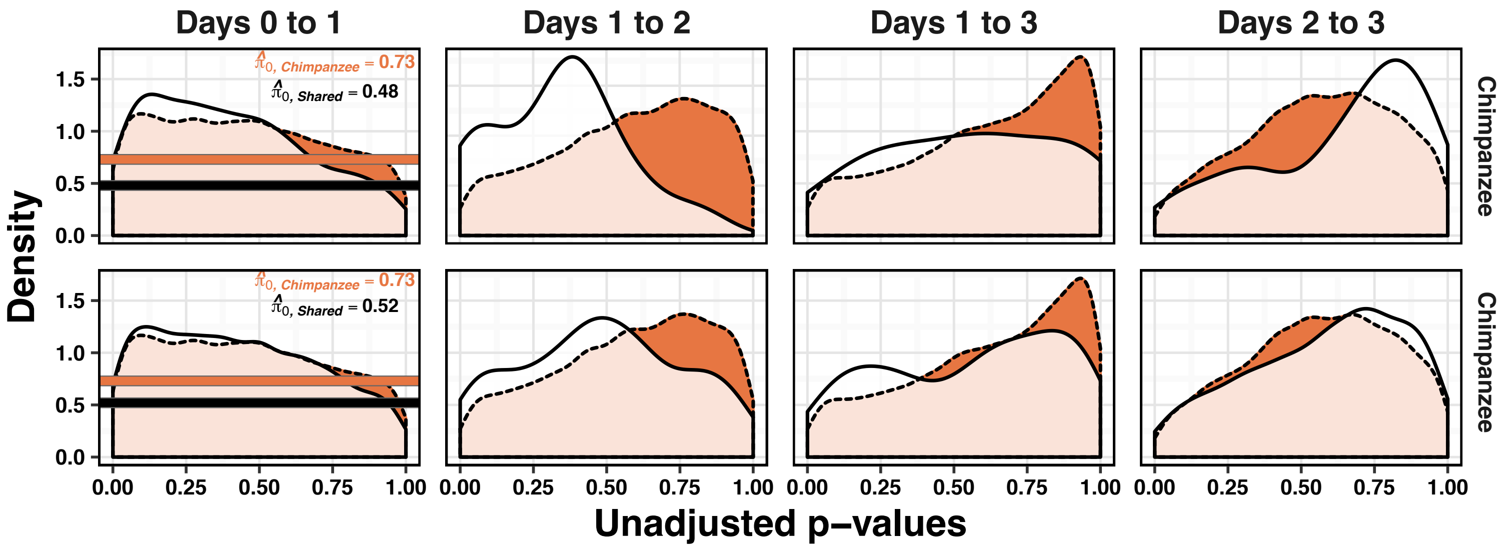

S15B.

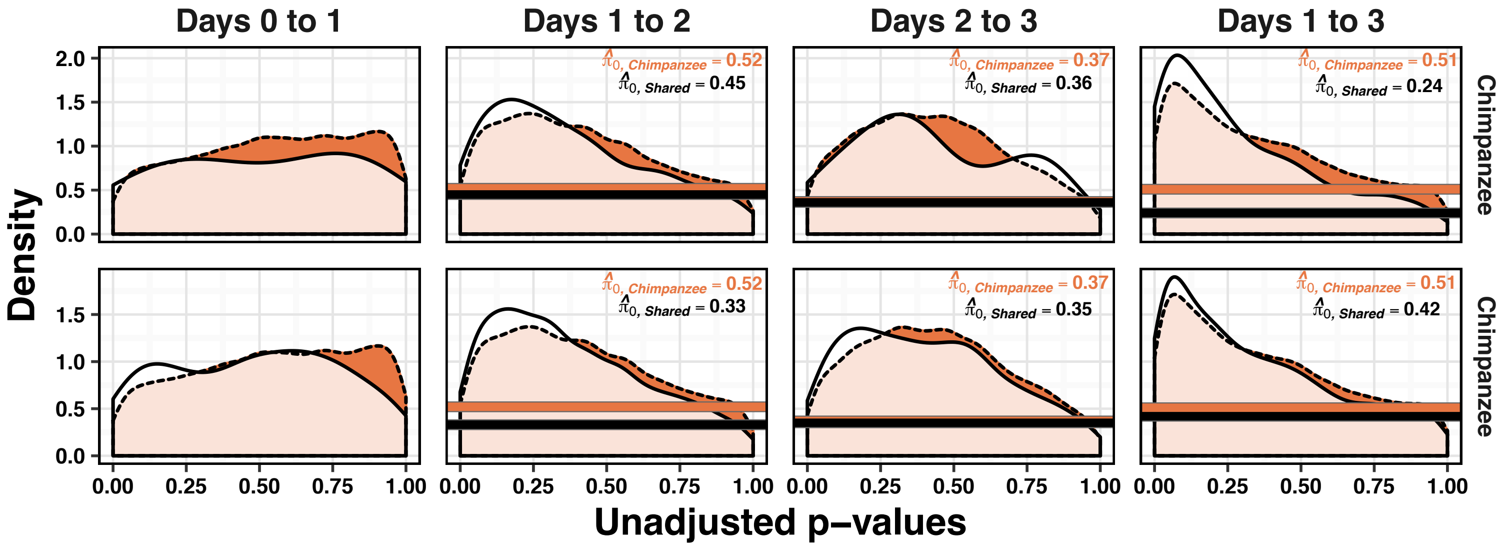

Figure S15: The patterns of change of variation in gene expression are robust with respect to P value cutoff in the chimpanzee samples. $\hat{\pi}_0$ is the estimated proportion of null tests in each distribution. A. P value distributions from F tests against the null hypothesis that there was no reduction in variation in gene expression levels as the samples progress along the time course for all genes from the chimpanzee samples (orange) and the genes in chimpanzees that were significant in the human samples (white, $P < 0.01$ on top, $P < 0.1$ on bottom row). $\hat{\pi}_0$ in the Days 1 to 2 test could not be computed in the top row. **B.** P value distributions of F tests against the null hypothesis that there was no increase in variation in gene expression levels as the samples progress along the time course for all genes from the chimpanzee samples (orange) and the genes in chimpanzees that were significant in the human samples (white, $P < 0.01$ on top, $P < 0.1$ on bottom row).

S16A.

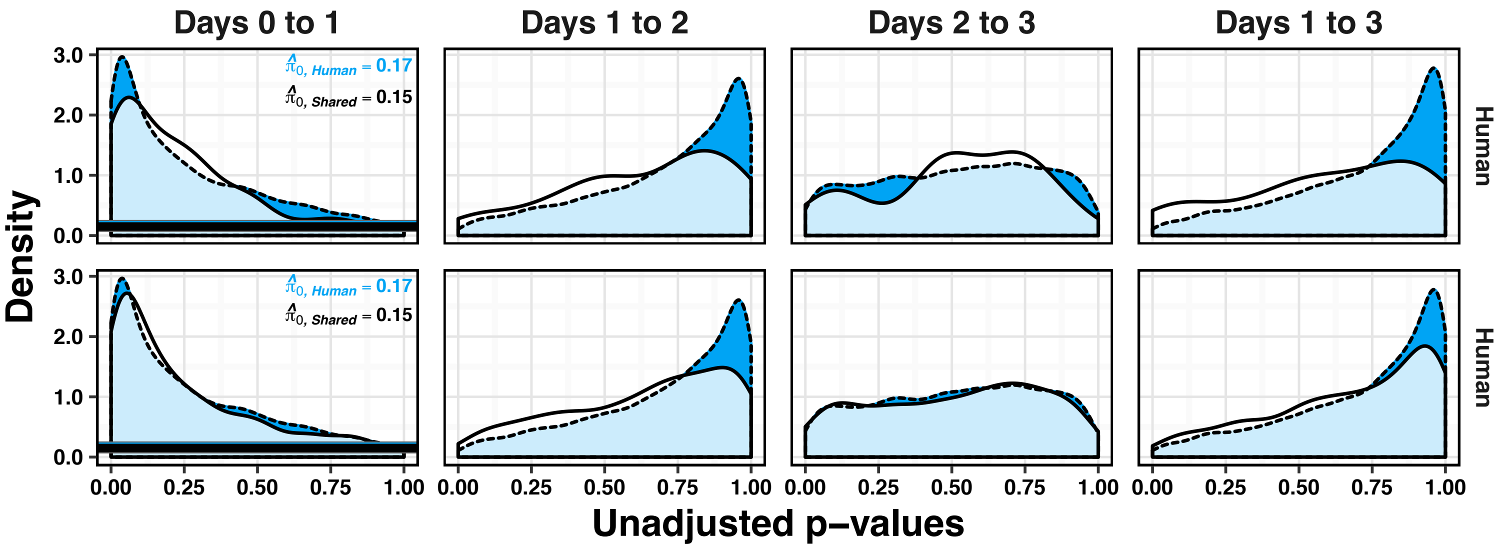

S16B.

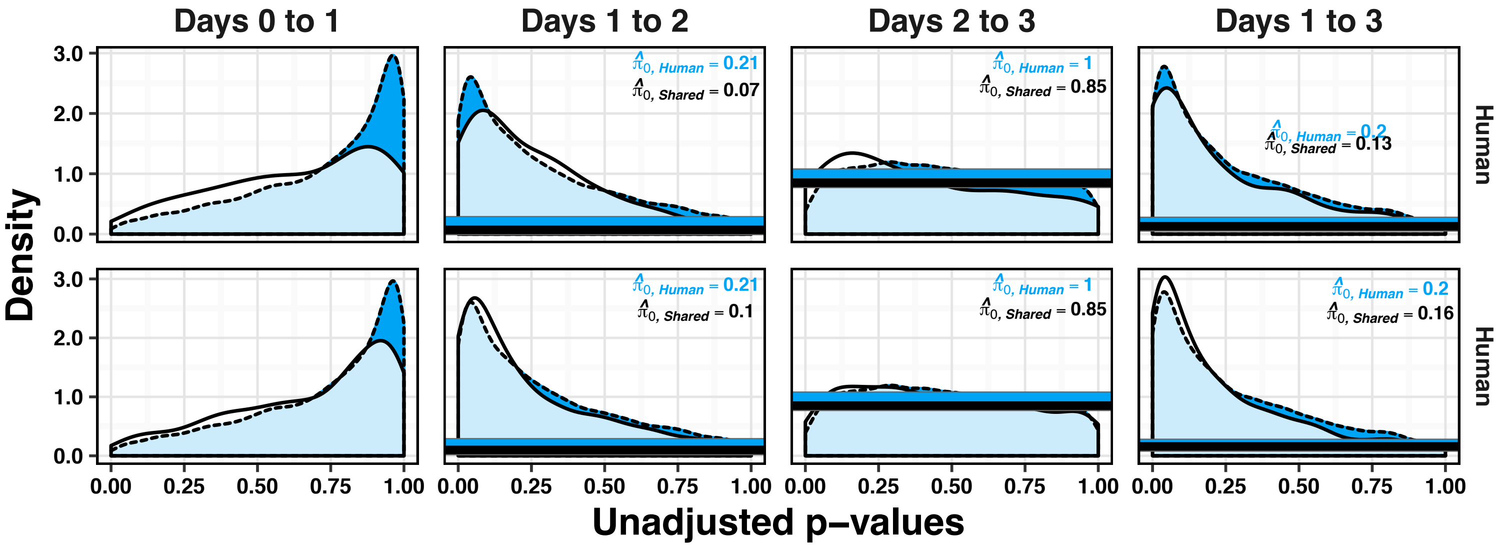

Figure S16: The patterns of change of variation in gene expression are robust with respect to P value cutoff in the human samples. $\hat{\pi}_0$ is the estimated proportion of null tests in each distribution. **A.** We plotted the P value distributions of F tests against the null hypothesis that there was no reduction in variation in gene expression levels as the samples progress along the time course for all genes from the human samples (blue) and the genes in humans that were significant in the chimpanzee samples (white, $P < 0.01$ on top, $P < 0.1$ on bottom row). **B.** Next, we plotted the P value distributions of F tests against the null hypothesis that there was no increase in variation in gene expression levels as the samples progress along the time course for all genes from the human samples (blue) and the genes in humans that were significant in the chimpanzee samples (white, $P < 0.01$ on top, $P < 0.1$ on bottom).

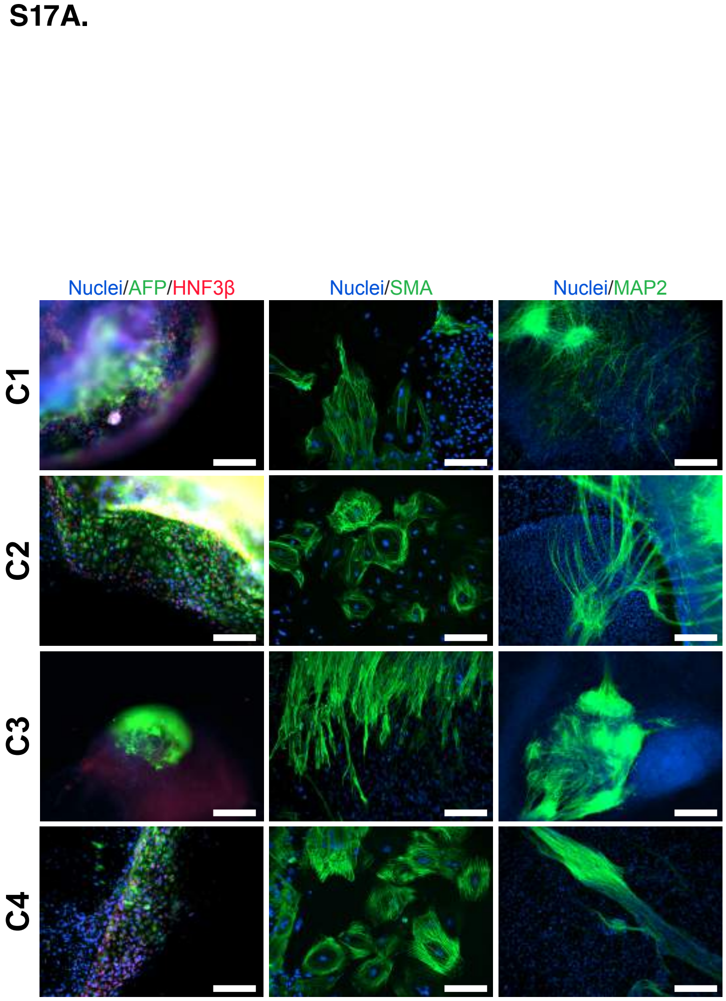

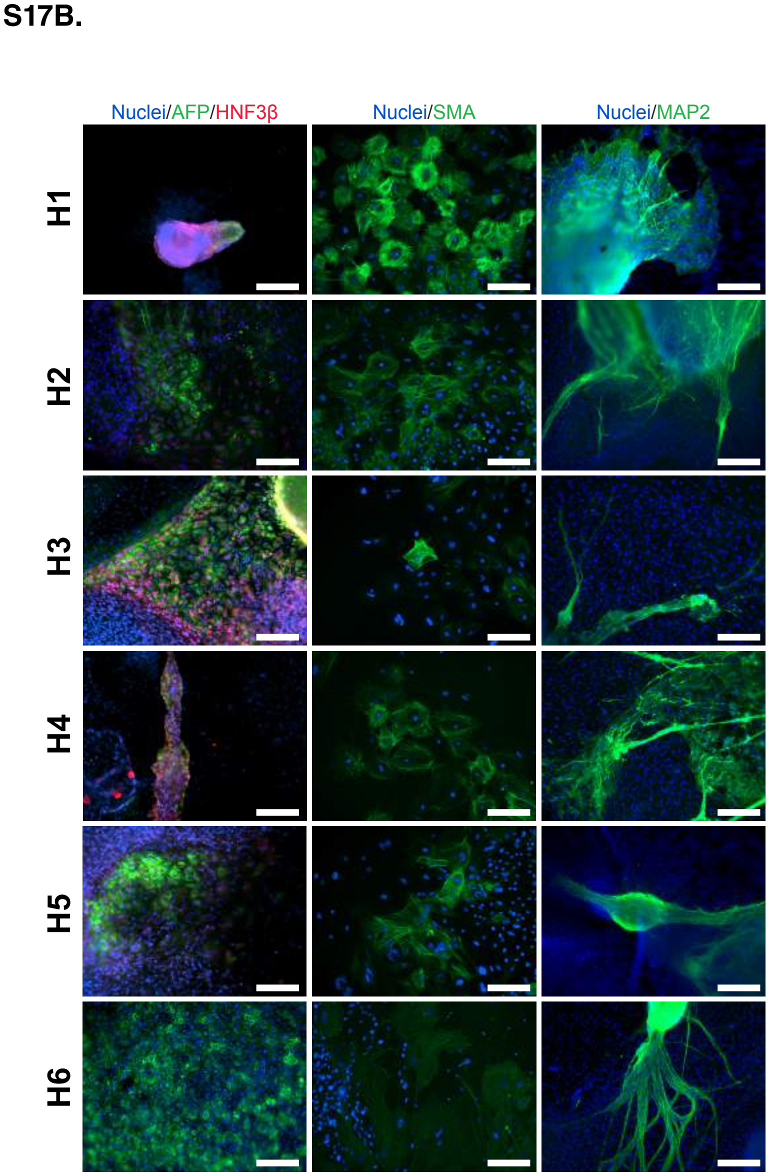

Figure S17: Assay of pluripotency for iPSC lines used in this study. A. Immunocytochemistry (ICC) staining of spontaneously differentiated embryoid bodies for chimpanzee iPSC lines.
B. Immunocytochemistry (ICC) staining of spontaneously differentiated embryoid bodies for human iPSC lines, antibodies identifying celltypes derived from the three germ layers as indicated.Scale bar: 200 µm.

S18A.

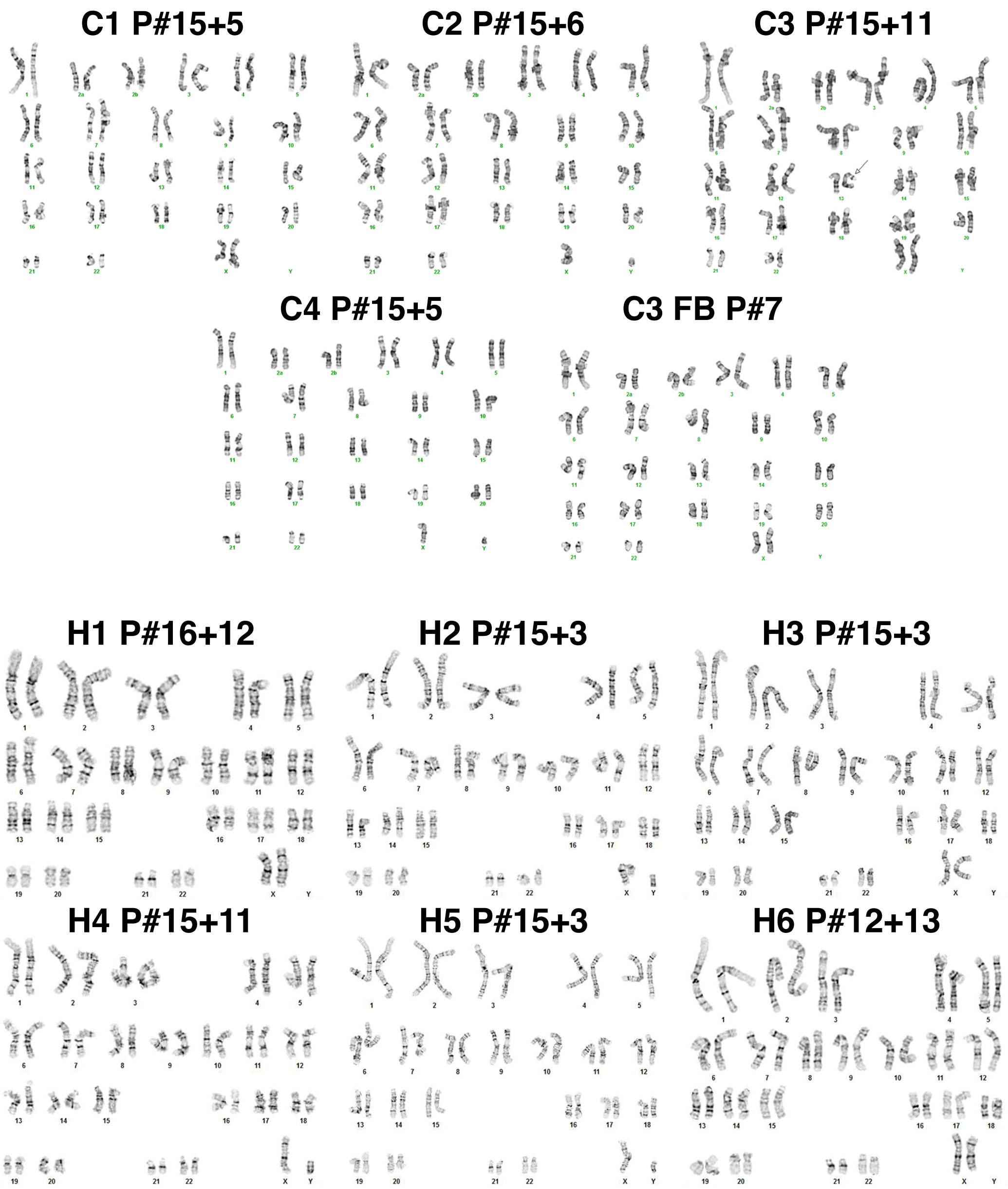

S18B.

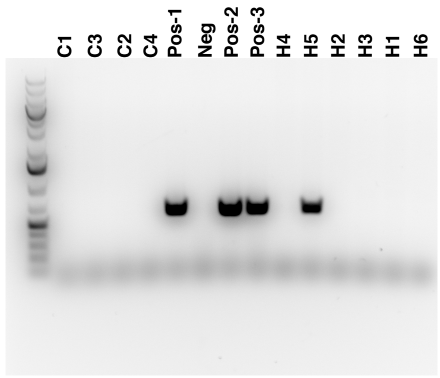

Figure S18: A. Karyotypes for human and chimpanzee iPSC lines. We identified additional bands in the p-arms of one chromosome 13 homolog and one chromosome 18 homolog for chimpanzee iPSC line C3. Thus we tested the source fibroblast line (C3 FB) to determine that these polymorphisms were normal polymorphisms and not formed de novo as a result of reprogramming. **B.** PCR gel to test for exogenous episomal reprogramming vectors in all iPSC lines used for this study. Pos indicates positive controls,Neg is a negative control. Human line H5 demonstrates a clear positive result for reprogramming plasmid.
