# Supplementary material for: A comparative study of endoderm differentiation in humans and chimpanzees
Source: Genome Biol. 2018 Oct 15;19:162. doi: 10.1186/s13059-018-1490-5 (PMC6191992; doi:10.1186/s13059-018-1490-5)
Supplement: Supplementary file 4 — Additional text and methods. Provides additional information about the Joint Bayesian analysis and a correlation-based method. (DOCX 32 kb) [file 13059_2018_1490_MOESM4_ESM.docx]

**Additional file 4**

**Text 1**

**Joint Bayesian analysis provides relatively consistent estimates of the conservation of gene expression patterns**

As stated in the main text, the joint modeling technique Cormotif [1] leveraged expression information shared across time points to identify the most common temporal expression patterns. In the main paper, we estimated the degree of conservation of trajectories based on a model with 8 correlation motifs to be 75%. When we choose the most likely model with 7 correlation motifs instead of 8 and used the same process to calculate the estimate as in the main text, this percentage rose slightly to 80% (Additional file 2: Figure S8B).

We also calculated the degree of conservation of gene expression trajectories using all 10,304 genes. We did so by using other values generated by Cormotif, the posterior probabilities of differential expression (DE) for every gene at each state transition in each species (e.g. the posterior probability of a given gene being DE in human samples between days 0 and 1). We calculated the percentage of genes with similar posterior probabilities of differential expression (≤ 0.20) across species at each comparison along the trajectory (e.g. a difference of ≤ 0.20 between the posterior probabilities of DE in the human and chimpanzee samples from days 0 to 1, as well as from day 1 to 2 and from day 2 to 3). The results suggested relatively strong conservation of gene expression trajectories across species, as 67% of the 10,304 genes tested had similar posterior probabilities of DE across species at all three comparisons. This percentage was similar (71%) when we calculated this estimate with 7 correlation motifs instead of 8. Overall, our estimates of the degree of conservation using analysis from Cormotif were relatively consistent (range: 67%-80%).

**The estimates of the conservation of gene expression patterns are relatively consistent when using a joint Bayesian method and a correlation-based method**

The joint modeling approach Cormotif allowed us to utilize the multiple data point structure of our data. There are also some limitations to this method, however. For example, genes are grouped based on the probability that they are DE but the approach does not take into account directionality. Therefore, genes with increased and decreased expression could theoretically be included in the same correlation motif. Furthermore, Cormotif ultimately relies on pairwise comparisons, albeit among all data points. To address some of these considerations, and to ensure that our results are robust we used an additional approach to analyze the data.

We utilized an approach specifically designed to group genes with similar expression patterns for time-series studies with 8 or fewer time points, called Short Time-series Expression Miner (STEM) [2, 3]. After filtering genes that only showed small changes in expression as recommended by the program (see Supplementary Methods below), 3940 genes with relatively large changes in gene expression levels over the timecourse remained for analysis. In the human samples, 3260 of these genes clustered into 9 significant model profiles (Additional file 2: Figure S7B). (There were an additional 20 profiles that did not contain enough genes to reach significance.) 2846 were clustered into 6 significant model profiles in the chimpanzees (Additional file 2: Figure S7B). (There were 24 additional non-significant profiles for the chimpanzee samples.) All of the significant chimpanzee profiles are also significant profiles using human samples, highlighting the similarities of gene expression trajectories across the species. Supporting our assessment of the high degree of conservation, the 3 significant human profiles that did not reach significance using chimpanzee samples nevertheless contained the same ranking (by number of genes) in each species.

To determine the proportion of genes with conserved trajectories for this method, we analyzed all genes assigned to the same cluster (1685 genes). We then identified an additional 1020 genes in similar clusters (mean profile correlation = 0.73, see Supplementary methods below). Using this approach, we discovered 2705 genes (or 69% of the 3940 genes analyzed) with the same or a highly correlated trajectory across species. This high degree of conservation across the timecourse is very similar to the estimates provided by the joint Bayesian method.

We found that the STEM clustering frequently assigned known regulators or markers of the differentiation process to the same cluster. This increased our confidence in the results, as we had a prior expectation that these markers would be assigned to the same or similar profiles for both species. For example, *EOMES* and *MIXL1*, transcription factors essential for endoderm formation, are assigned to the same clusters across species (Additional file 2: Figure S7A). Furthermore, of all the developmental regulators and markers examined (a total of 34 gathered from literature) the majority (70%) are assigned to the same cluster in both species, and an additional 20% assigned to highly similar profiles (correlation ≥ 0.70). Most of these 34 genes (61%) are assigned to just two profiles, suggesting that these profiles are likely the representative trajectories for developmental progression and drivers of endoderm specification.

**Methods**

**A correlation-based method designed for short time-series gene expression studies**

In order to classify gene expression trajectories across species, we used a Java program specifically designed for time course microarray data with a small number of time points called Short Time-series Expression Miner (STEM)[2, 3]. We used TMM-normalized log2(CPM) expression values averaged across technical replicates (n = 40) because it was unclear how we could both model expression with replicates and still use the program to complete analyses related to the overlapping trajectories across species. We note that the main paper used TMM- and cyclic loess- normalized log_2_CPM data, but the two datasets are highly correlated (r > 0.99).

Per the STEM manual’s suggestion for removing gene expression trajectories driven solely by technical noise, we prefiltered our set of 10,304 orthologous genes using a minimum difference of at least one log2 fold change between the minimum and maximum values (not necessarily across consecutive days). We also filtered out the genes that were in the bottom quantile of the within-species correlations. We chose a quantile cutoff rather than a value cutoff for each species because the chimpanzee samples were generally more highly correlated than the human samples. To ensure that the same genes were used for analysis, filtering was done outside of STEM using a custom R script and the union of the gene passing filtering criteria in either species was utilized (i.e., a gene only needed to pass inclusion criteria in one of the two species to be considered for further analysis). 3940 genes remained post-filtering and were used to first run the chimpanzee and human expression data separately (1 run each).

Using the Clustering Method option, we used the “STEM Clustering Method” and selected the options Maximum Number of Model Profiles = 125 and Maximum Unit Change in Model Profiles between the Time Points = 2. The former was selected so that we could still maintain a reasonable number of profiles (maximum of 125) and the number of significant model profiles was relatively robust to the maximums unit change in model profiles between time points.

Since we had previously filtered the data, when we reached the filtering stage in the program, we selected values in the STEM program that would not cause any new genes to be removed. Specifically, we used the following options: Maximum Number of Missing Values = 0, Minimum Expression Change = 0, Minimum Correlation between Repeats = -1. We also normalized the data so that the first time point had a gene expression level of 0 and all subsequent values would be log_2_ fold changes relative to the first time point.

We used the STEM package to calculate the correlation of each gene to each model profile and to assign each gene to the model profile with which it had the highest correlation. We noticed during early runs of this program using expression values from either species that there were many highly correlated model profiles which we felt would still be well represented but be more interpretable if they were combined into a single profile. Therefore, we selected the option to collapse highly correlated model profiles into a single representative model profile. After each of our 3260 genes were assigned to model profiles, we used the package to determine the significance of the number of genes assigned to each profile by a permutation test. We therefore set options Maximum Correlation = 0.9, Number of Candidate Model Profiles = 1,000,000, Number of Permutations per Gene = All permutations and Significance Level = 0.05 with Correction Method = Bonferroni for the assignment and test of significance steps.

Through this process, we acquired the lists of all model profiles with at least 1 gene assigned to it, the gene names assigned to each model profile, and which model profiles were significant in each species. We used the “overlap” option in STEM to determine which profiles were significant in both species or only significant in one species. To find genes that were significantly enriched in correlated clusters, the compare option was used within STEM using a maximum uncorrected intersection *P* value of 0.005 and only considering profile intersections of at least 20 genes.

**References**

1. Wei Y, Tenzen T, Ji H: **Joint analysis of differential gene expression in multiple studies using correlation motifs.** *Biostatistics* 2015, **16:**31-46.

2. Ernst J, Bar-Joseph Z: **STEM: a tool for the analysis of short time series gene expression data.** *BMC Bioinformatics* 2006, **7:**191.

3. Ernst J, Nau GJ, Bar-Joseph Z: **Clustering short time series gene expression data.** *Bioinformatics* 2005, 21.
